# Supplementary material for: Alkyl Derivatives of Perylene Photosensitizing Antivirals: Towards Understanding the Influence of Lipophilicity
Source: Int J Mol Sci. 2023 Nov 18;24(22):16483. doi: 10.3390/ijms242216483 (PMC10671050; doi:10.3390/ijms242216483)

Alkyl derivatives of perylene photosensitizing antivirals: towards understanding the influence of lipophilicity

Igor E. Mikhnovets [1,†], Jiří Holoubek [2,3,4,†], Irina S. Panina [1], Jan Kotouček [5], Daniil A. Gvozdev [6], Stepan P. Chumakov [1], Maxim S. Krasilnikov [1,7], Mikhail Y. Zhitlov [1,7], Evgeny L. Gulyak [1], Alexey A. Chistov [1], Timofei D. Nikitin [1], Vladimir A. Korshun [1], Roman G. Efremov [1], Vera A. Alferova [1], Daniel Růžek [2,3,4], Luděk Eyer [2,3,4] and Alexey V. Ustinov [1,*]

1 Shemyakin-Ovchinnikov Institute of Bioorganic Chemistry, Miklukho-Maklaya 16/10, 117997 Moscow, Russia
2 Laboratory of Emerging Viral Diseases, Veterinary Research Institute, Hudcova 296/70, CZ-621 00 Brno, Czech Republic
3 Institute of Parasitology, Biology Centre of the Czech Academy of Sciences, Branišovská 1160/31, CZ-370 05 České Budějovice, Czech Republic
4 Department of Experimental Biology, Faculty of Science, Masaryk University, CZ-62500 Brno, Czech Republic
5 Department of Pharmacology and Toxicology, Veterinary Research Institute, Hudcova 296/70, CZ-621 00 Brno, Czech Republic
6 Department of Biology, Lomonosov Moscow State University, Leninskie Gory 1-12, 119234 Moscow, Russia
7 Department of Chemistry, Lomonosov Moscow State University, Leninskie Gory 1-3, 119991 Moscow, Russia
* Correspondence: austinov@yandex.ru
† These authors contributed equally to this work.

Supplementary information

S1. Isomers ratio in mixture of 5a is 34% to 66% of 10-butyl,3-ethynylperylene to 9-butyl,3-ethynylperylene.

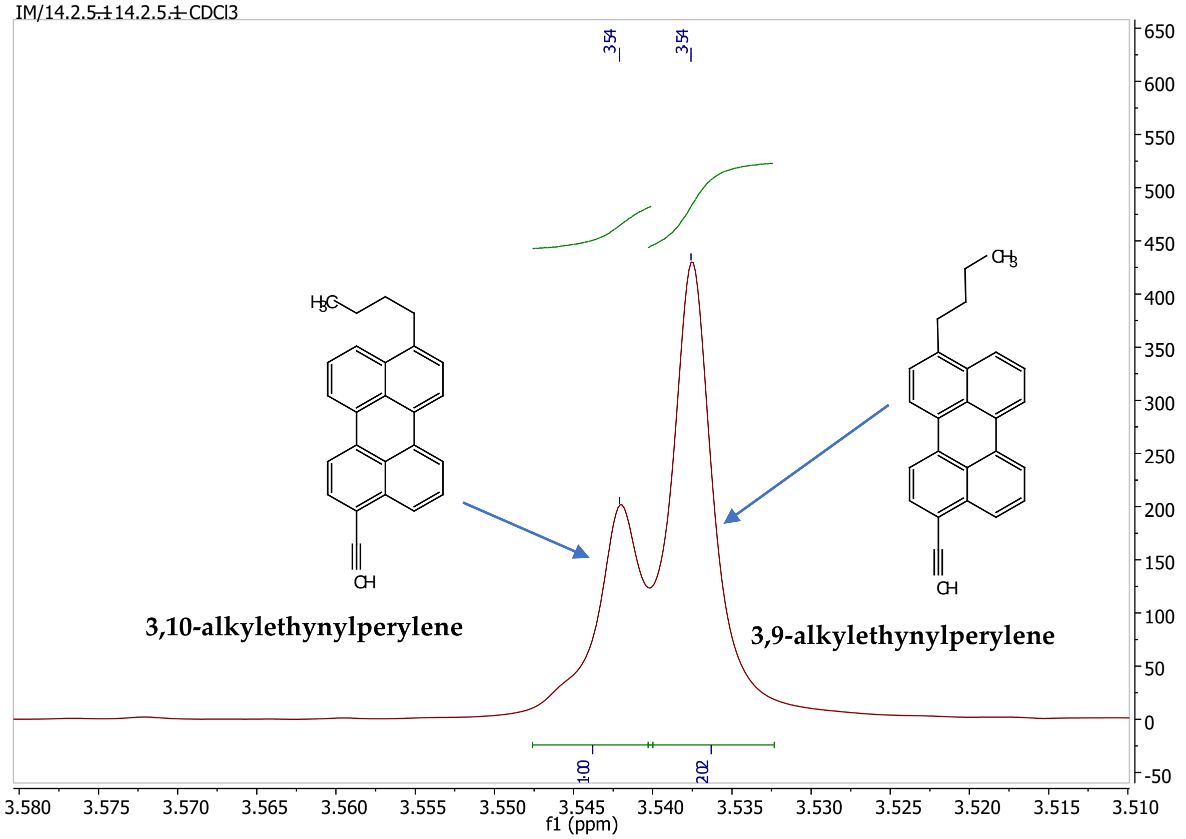

Figure S1. Mixture of isomers in compound **5** contains 34% of 3,10-alkylethynylperylene and 66% of 3,9-alkylethynylperylene based on ^{1}H NMR data. Terminal ethynyl proton of compound 3,9-alkylethynylperylene has slightly more electronic density on it than corresponding proton of 3,10-alkylethynylperylene. Therefore, signal in ^{1}H NMR spectra of 3,9-alkylethynylperylene has a slightly lower chemical shift than corresponding proton of 3,10-alkylethynylperylene.

S2. Molecular dynamics simulations

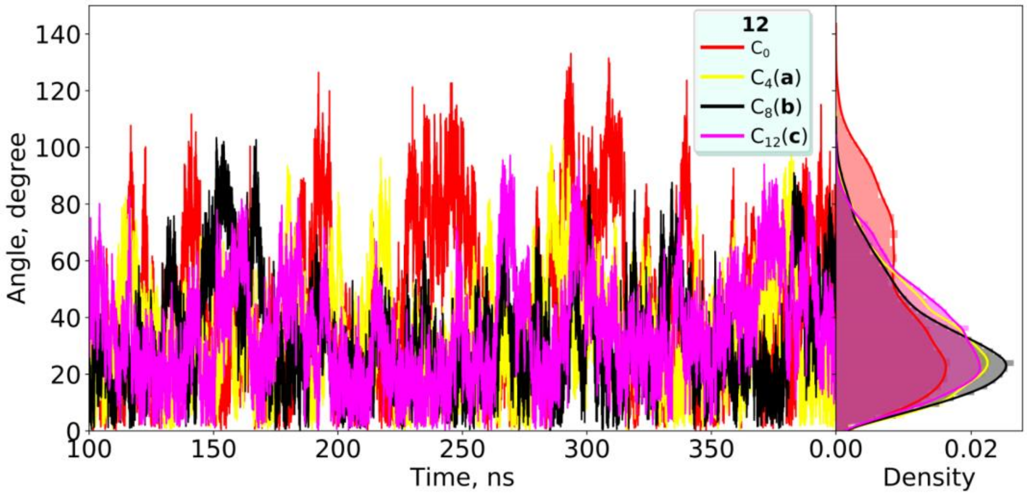

Figure S2-1. Dynamics of the tilt angle for the set of compounds 12 (C_0-C_{12}) in the course of MD simulations. The tilt angle values as a function of MD time (*left*) illustrate the lifetime of the major states. Probability density functions for the tilt angle distributions are shown *on the right*. One

representative MD trajectory for each alkyl-derivative is shown starting from 100-ns simulation time, when all compounds are embedded into bilayer.

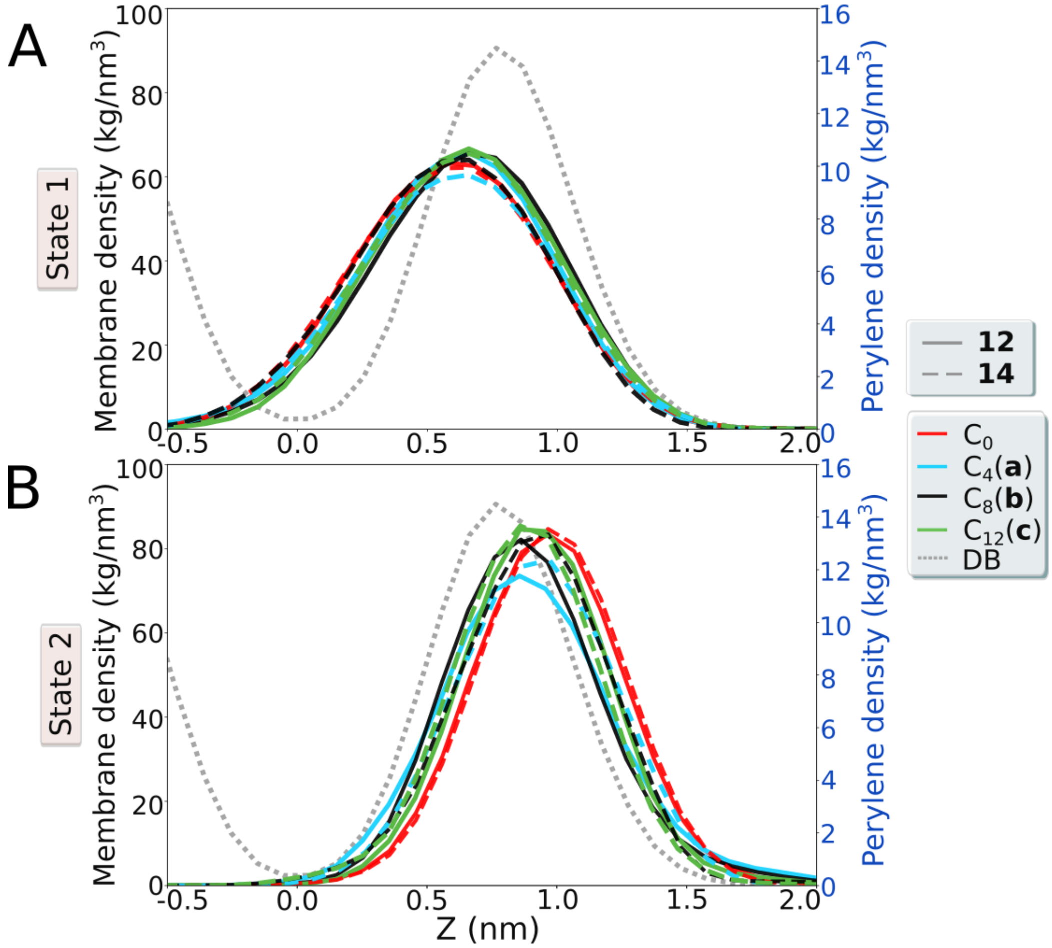

Figure S2-2. Dissimilar positioning of two major states of perylene derivatives in bilayer found in MD simulations. COM density profiles of the membrane-embedded perylene group (*right axis*) and lipids' double bond (DB; *grey dotted line; right axis*) of compounds in **state 1** (A) and **state 2** (B) relative to the bilayer center (Z = 0). The *solid lines* represent derivatives of compound **12**, and the *dashed lines* represent derivatives of compound **14**. In state 1, all molecules are located at a similar depth in the bilayer, whereas in state 2, the molecules without an alkyl group "float" closer to the surface.

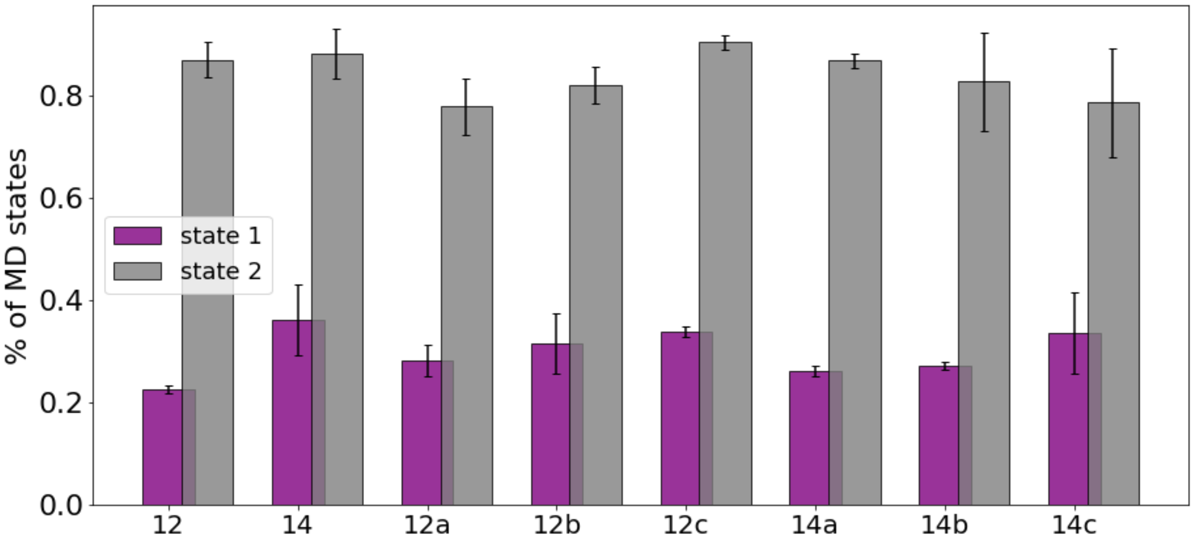

Figure S2-3. Water accessibility of the perylene group in lipid bilayer in the course of MD. The drastic difference between the number of frames with solvent-accessible perylene group in state 1 and state 2 common to all compounds.

S3. Antiviral activity towards VSV

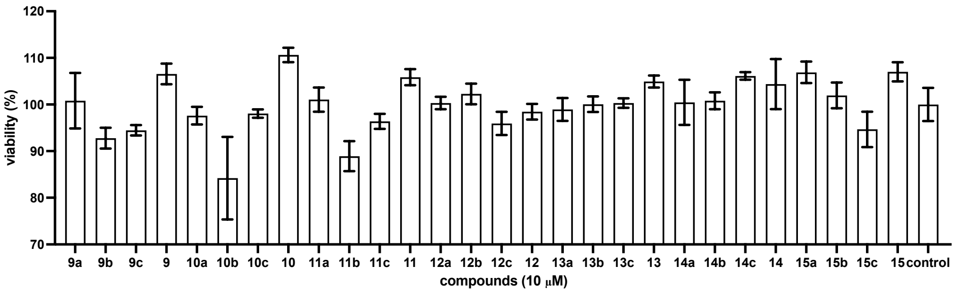

Figure S3-1. Cytotoxicity of the tested compounds towards HEK-293T cells at concentrations of 10 μM.

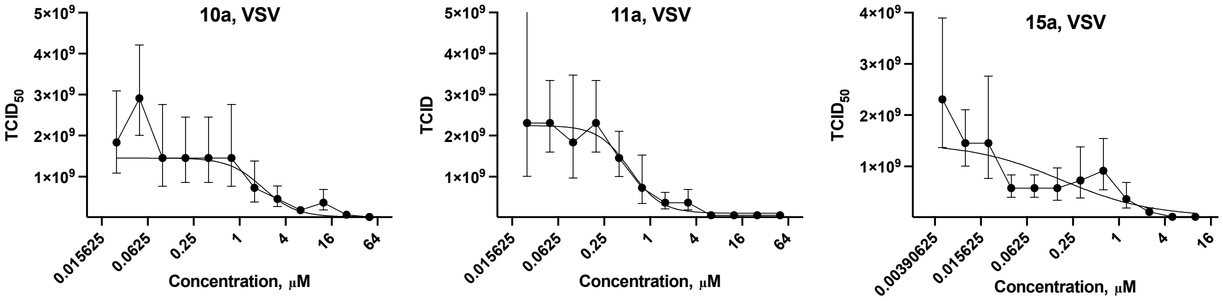

Figure S3-2. Titer reduction of VSV for compounds 10a, 11a and 15a assesed in HEK-293T cells. Initial concentrations were set at 50μM for compounds 10a and 11a, compound 15a started at 10 μM. For EC_{50} computationsinvolving compounds 10a and 15a, the top and bottom values were 1.45 x 10^9 and 1.8 x 10^8, respectively.

S4. NMR spectra of compounds 9-15a-c.

^{1}H NMR spectrum of **9a**

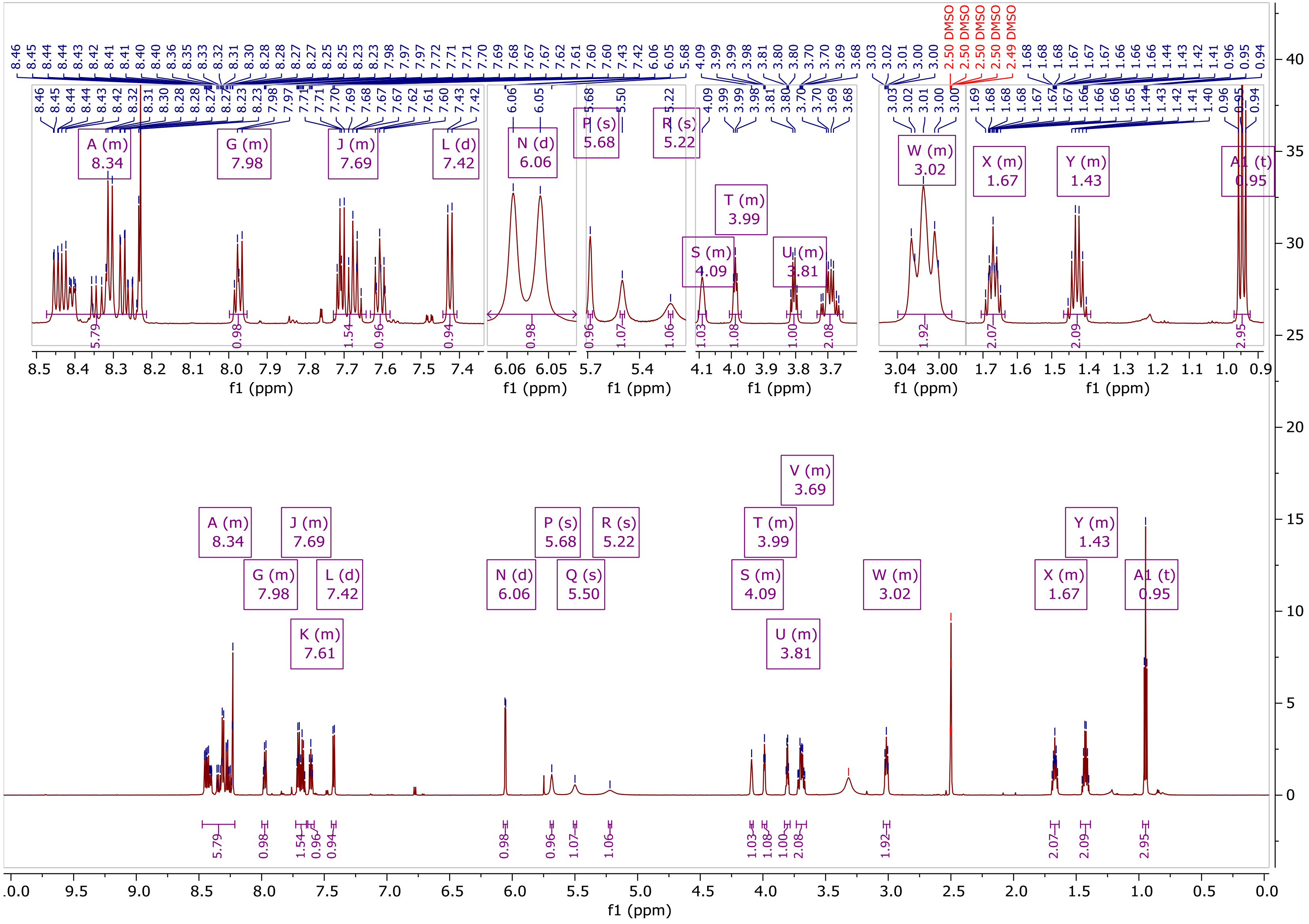

^{13}C NMR spectrum of **9a**

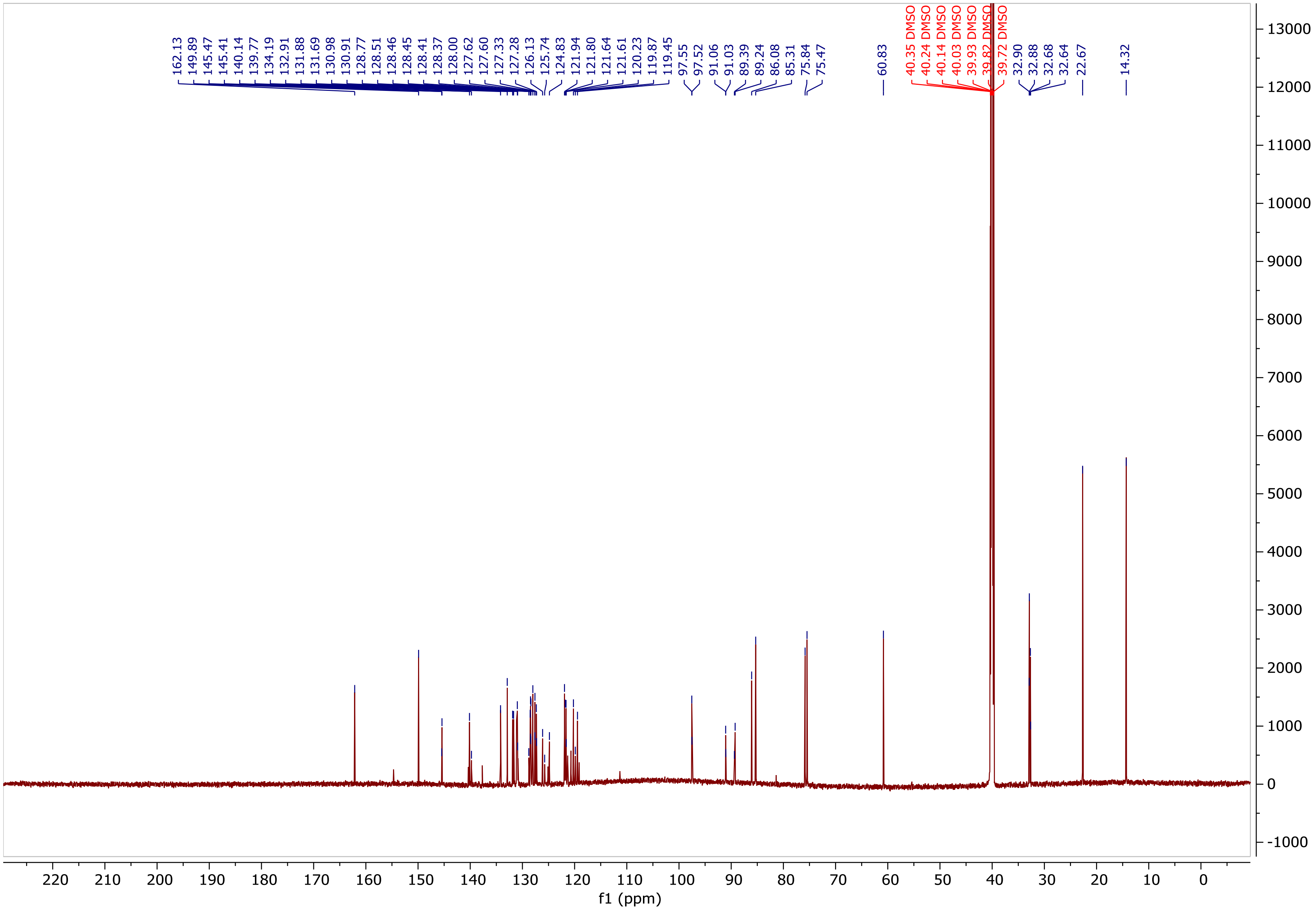

^{1}H NMR spectrum of **10a**

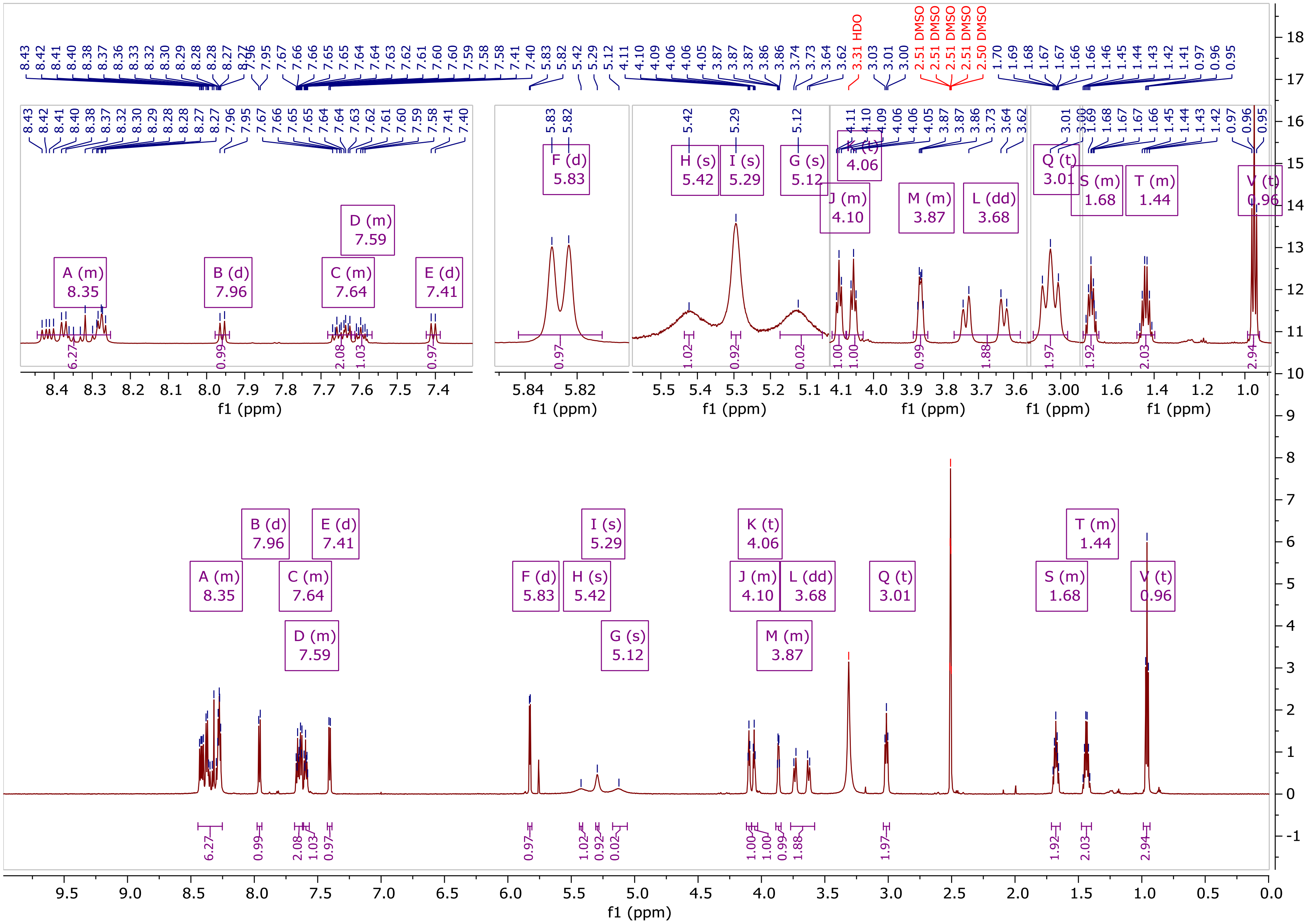

^{13}C NMR spectrum of **10a**

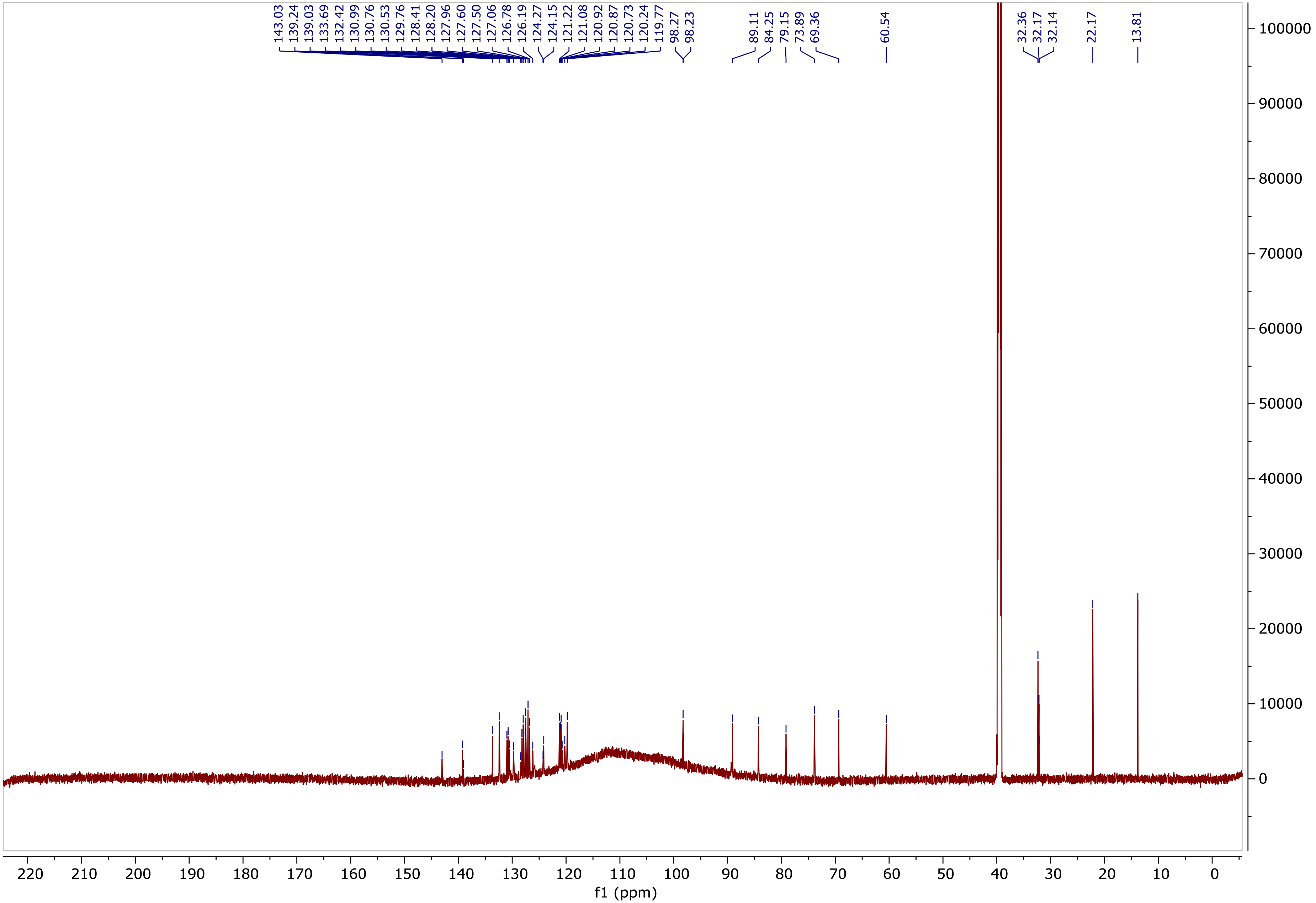

^1H NMR spectrum of **11a**

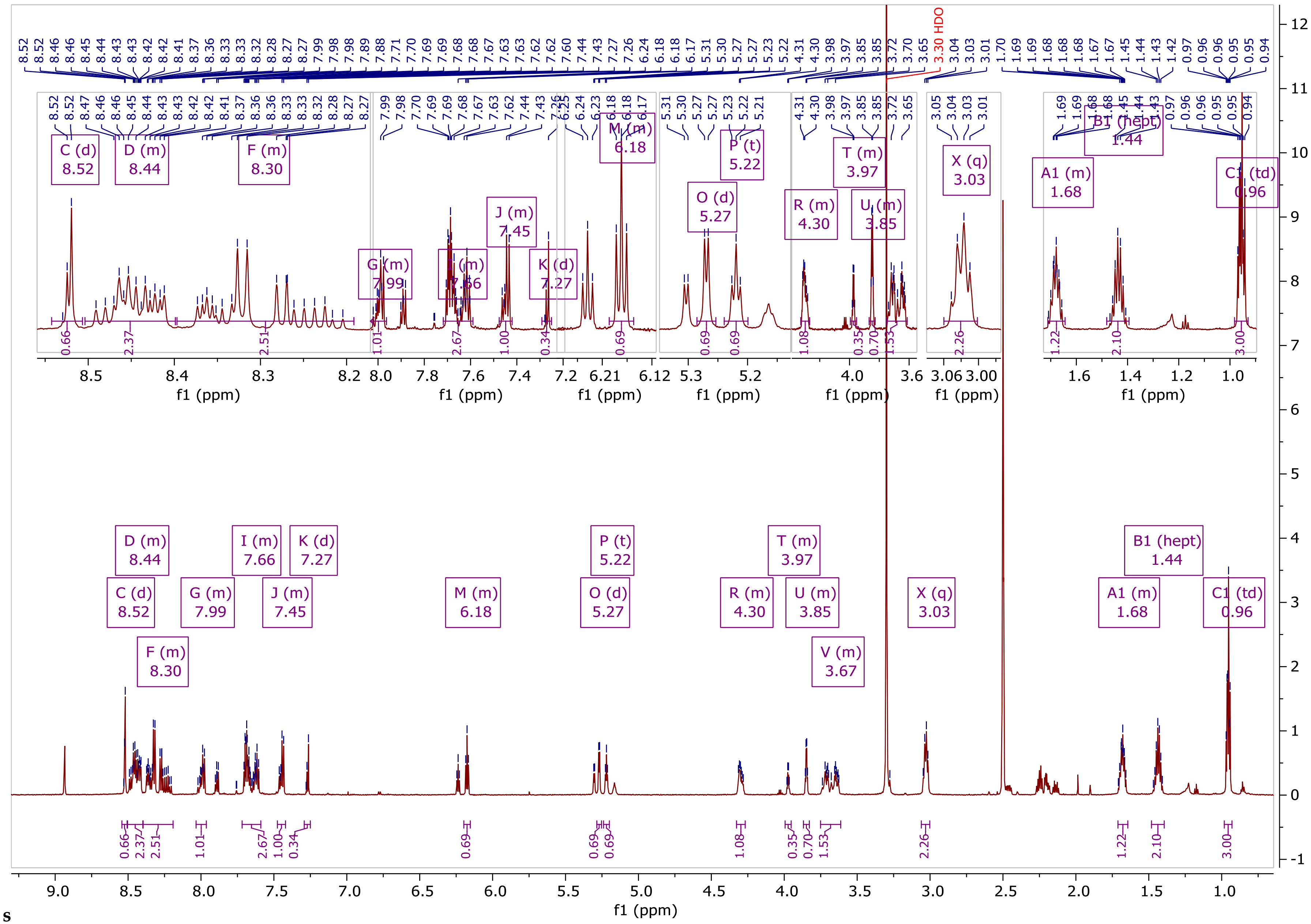

S

^{13}C NMR spectrum of **11a**

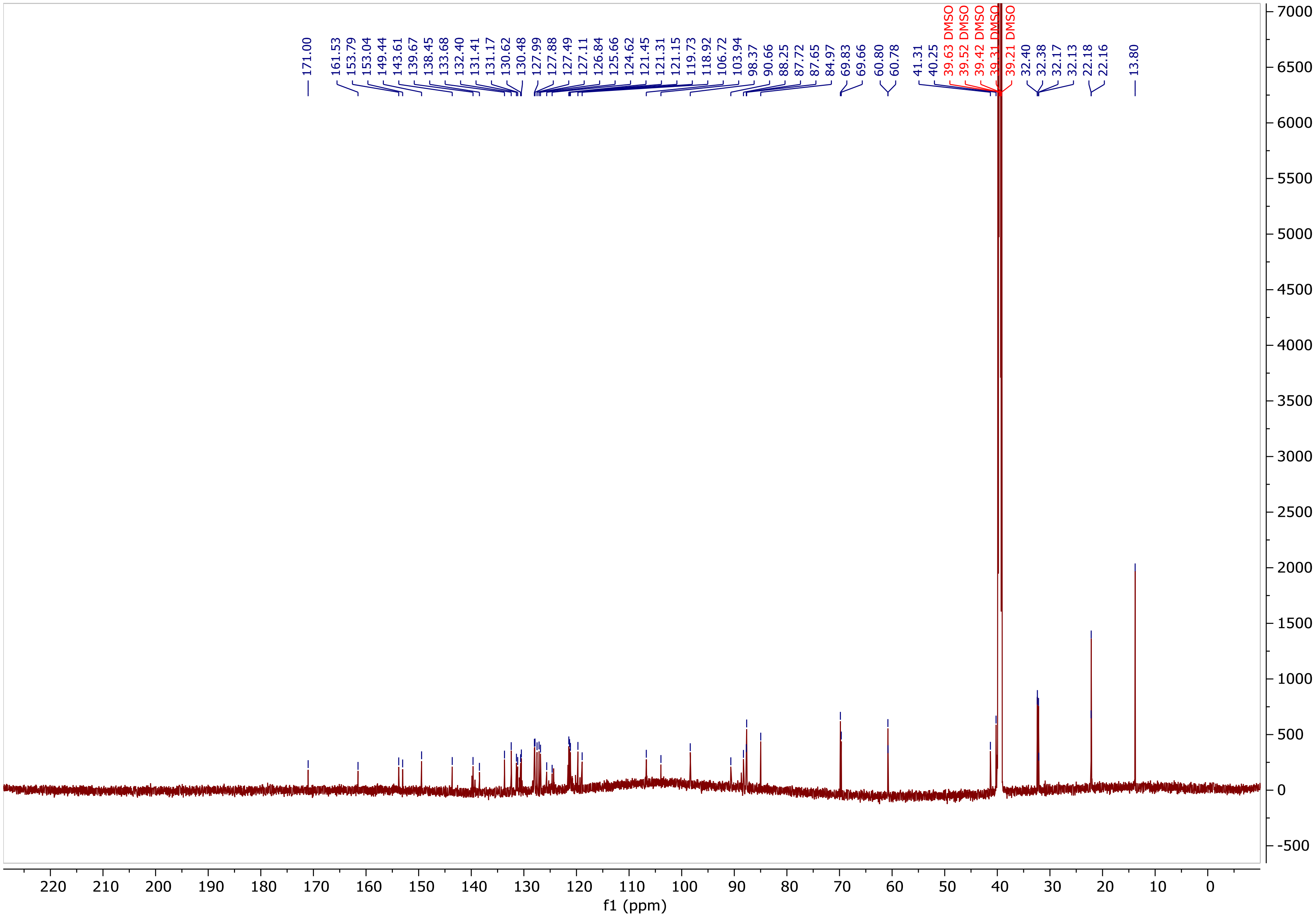

^{1}H NMR spectrum of **12a**

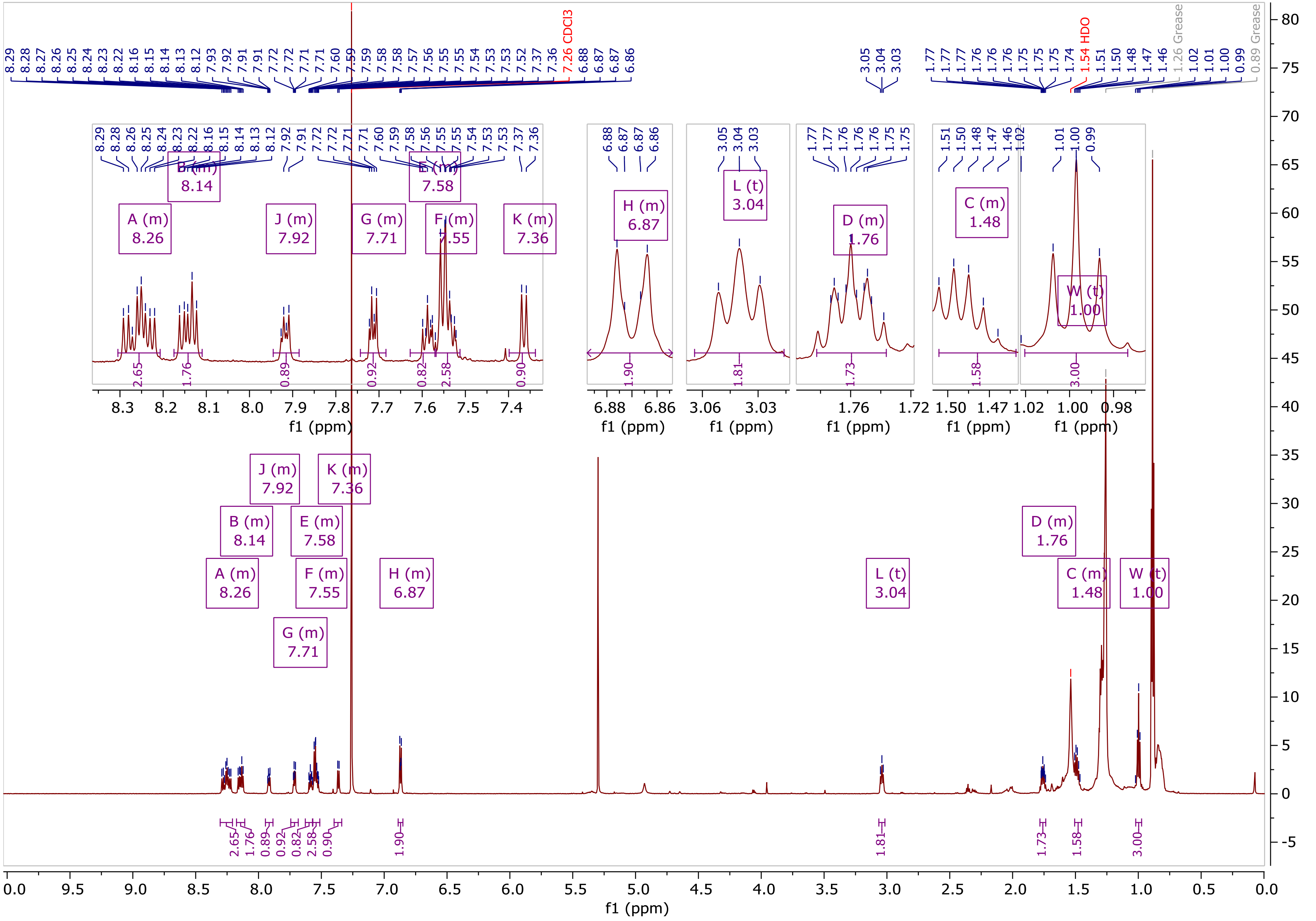

^{13}C NMR spectrum of **12a**

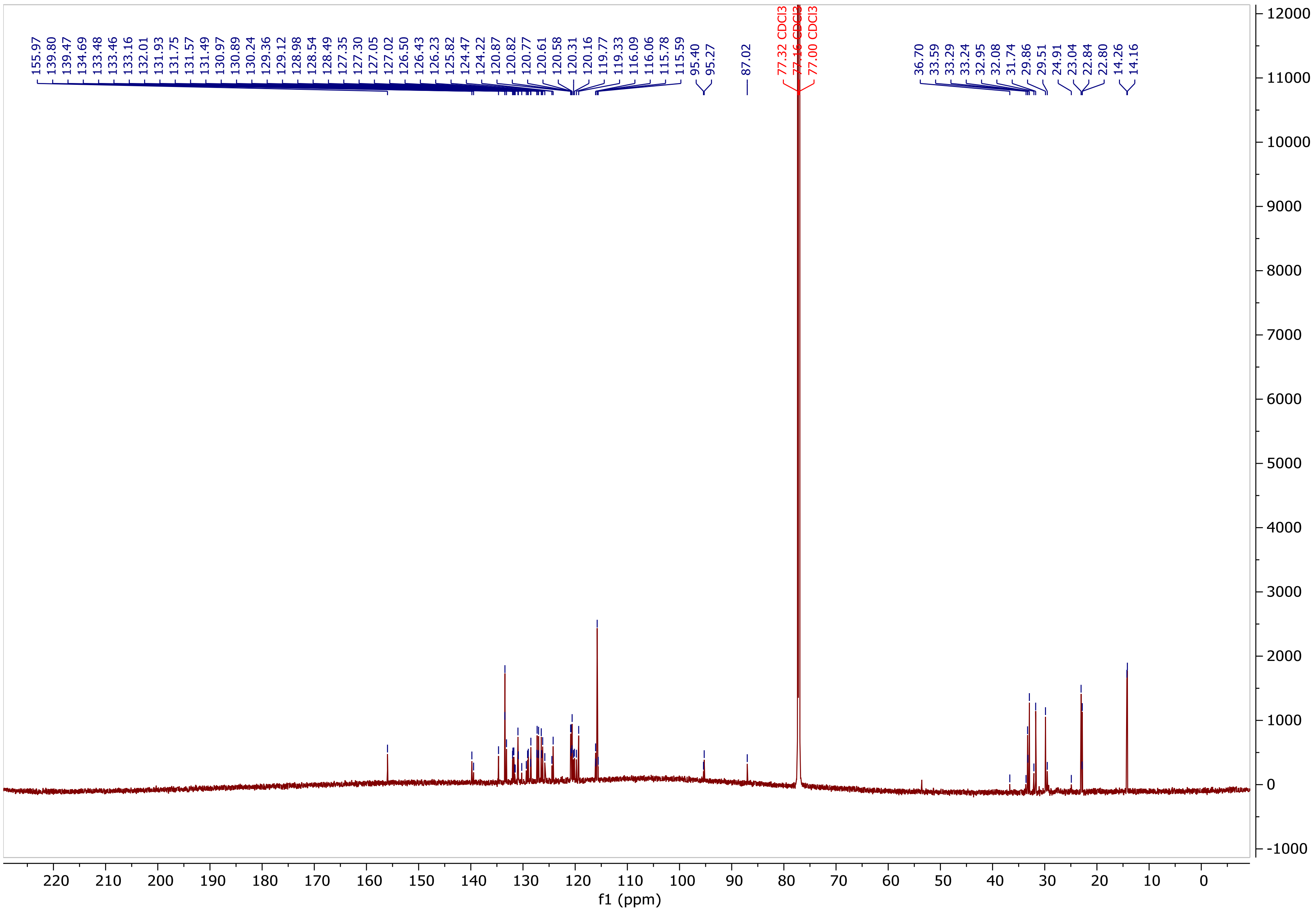

^{1}H NMR spectrum of **13a**

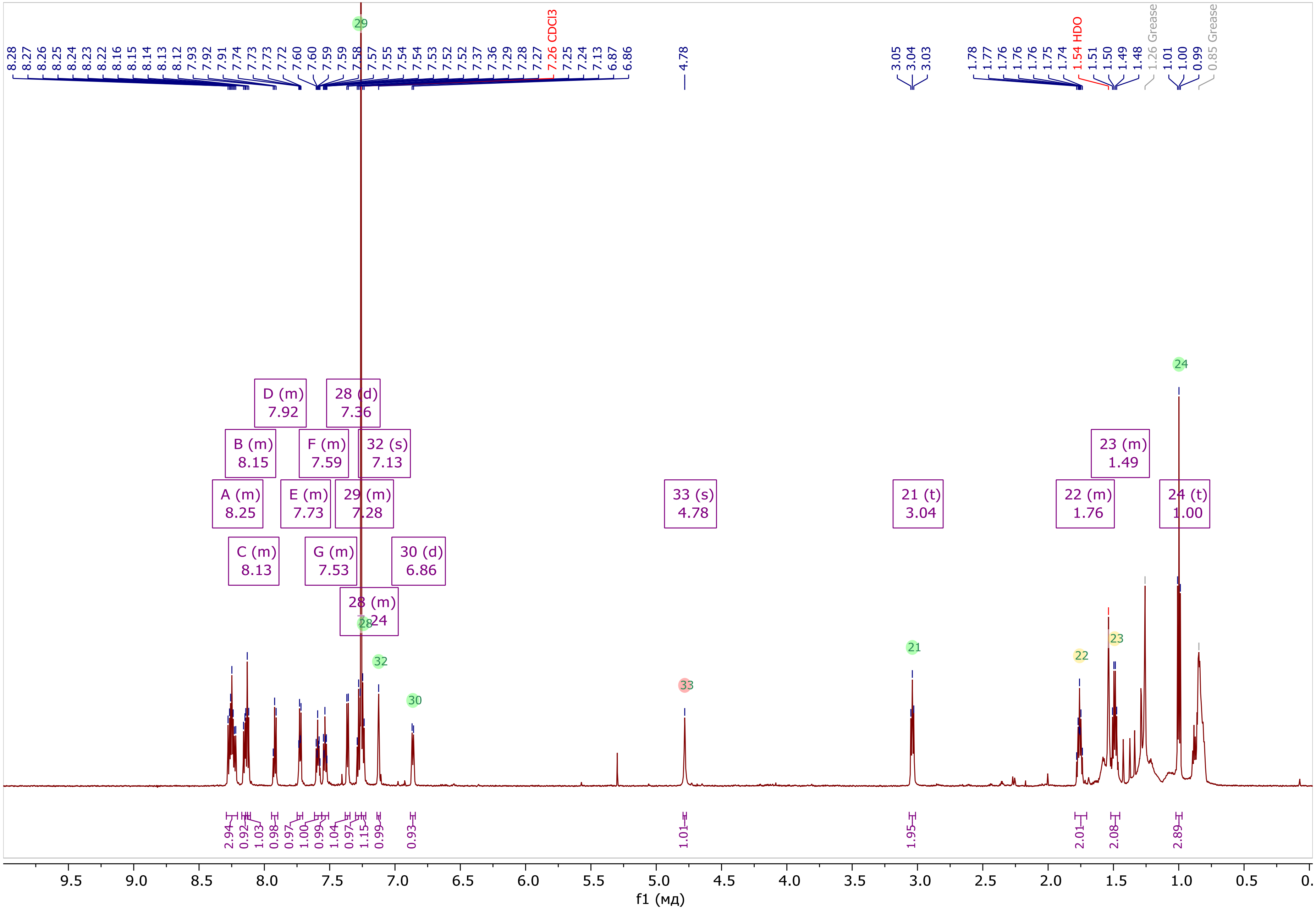

^{13}C NMR spectrum of **13a**

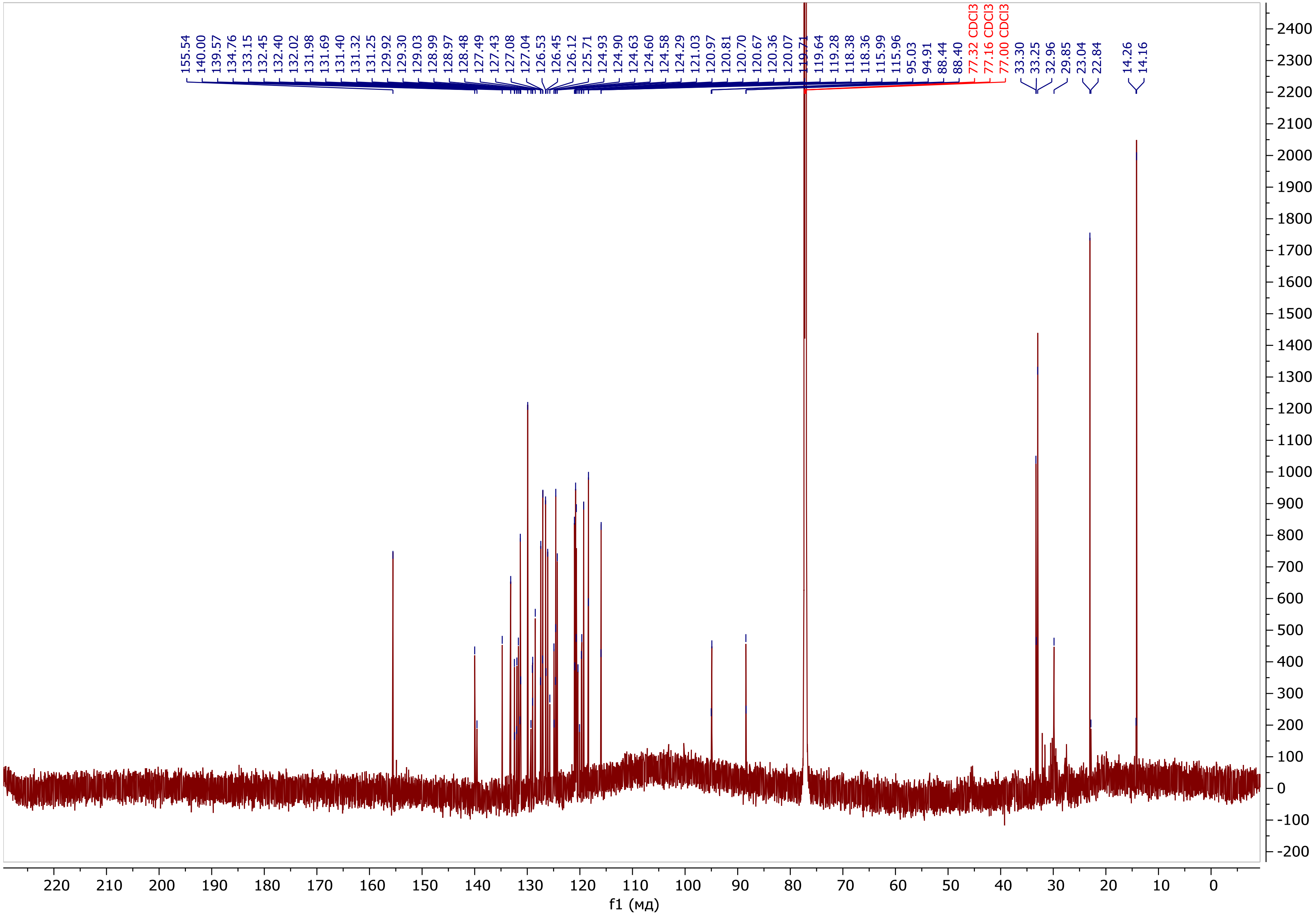

^{1}H NMR spectrum of **14a**

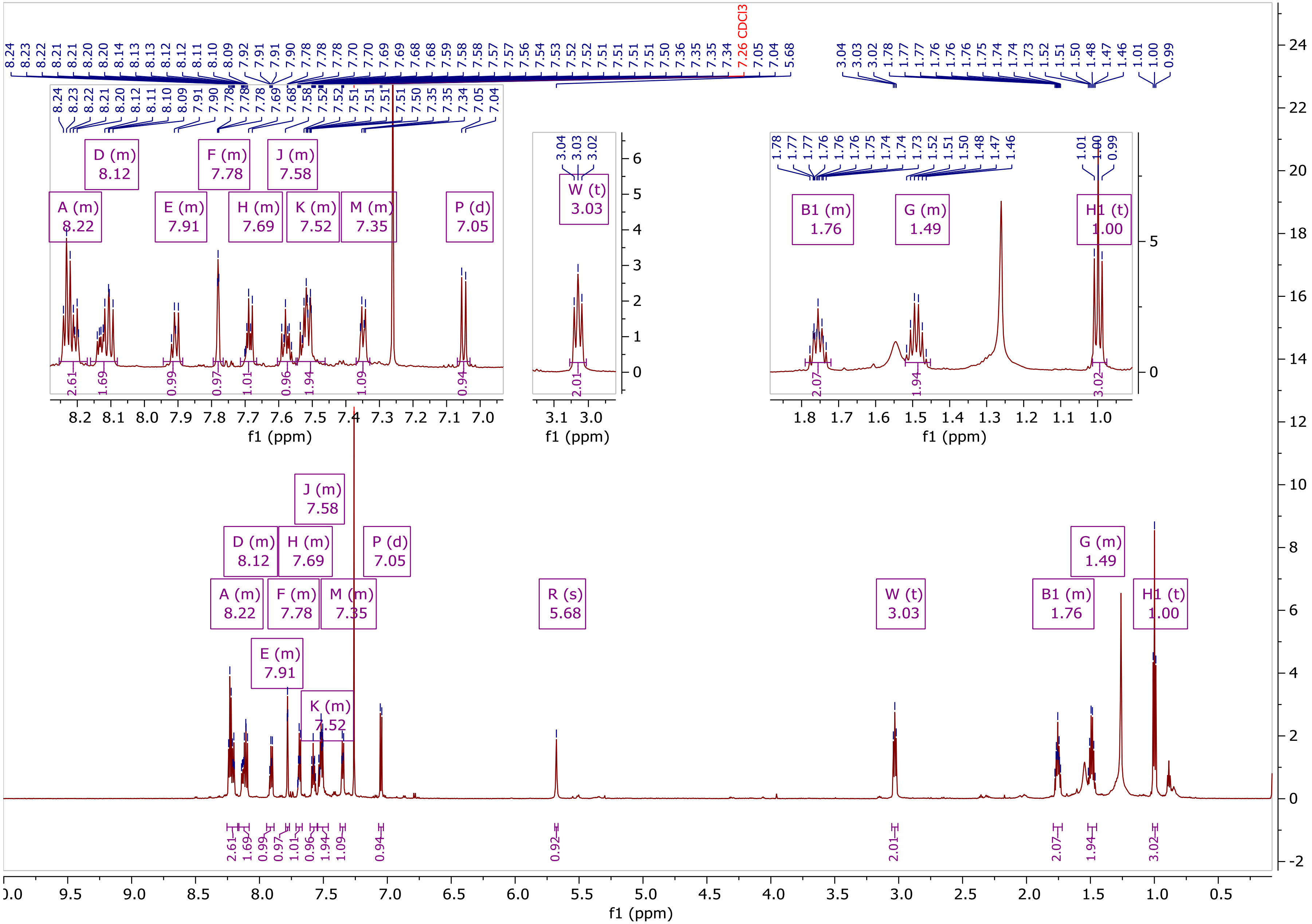

^{13}C NMR spectrum of **14a**

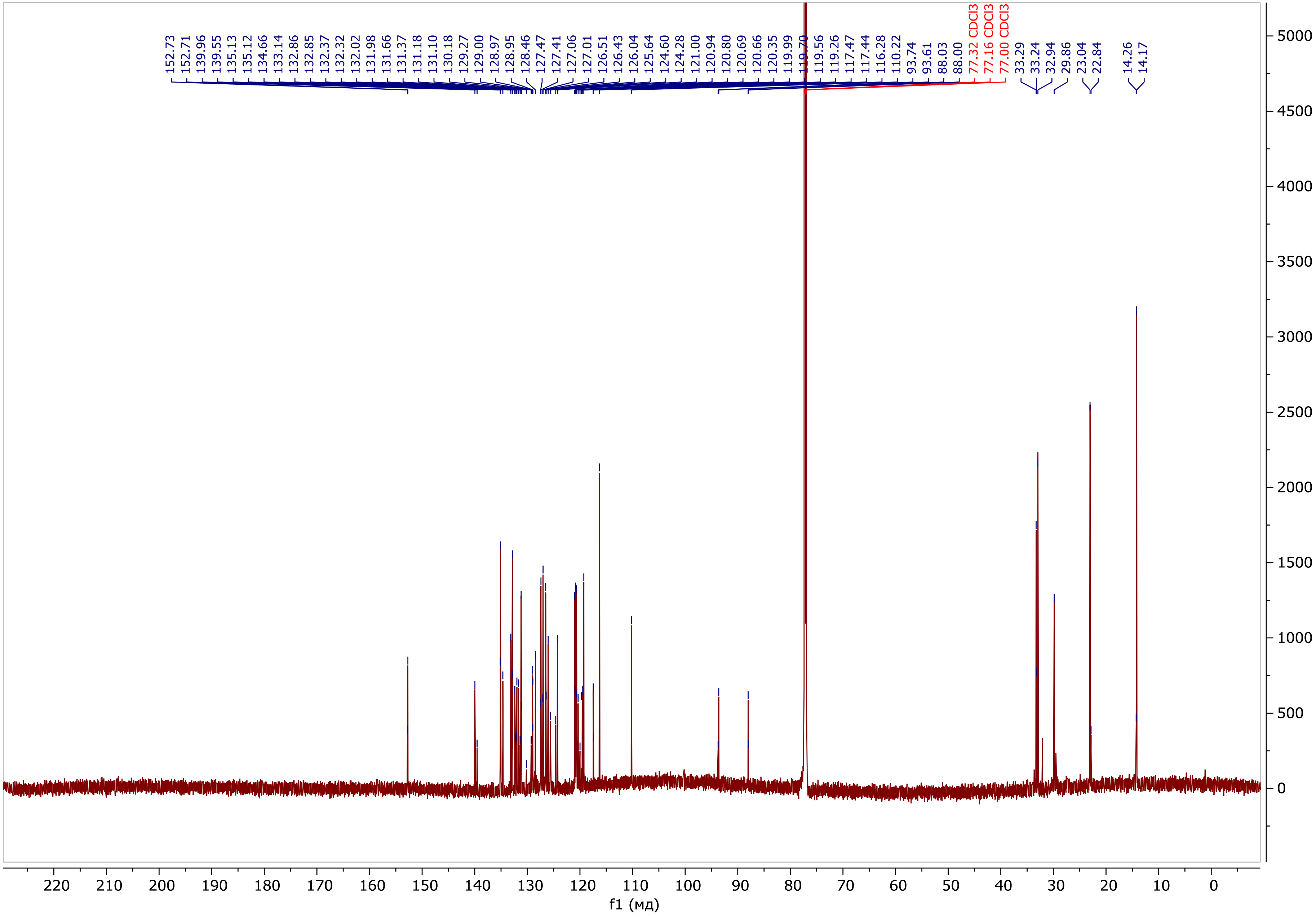

^{1}H NMR spectrum of **15a**

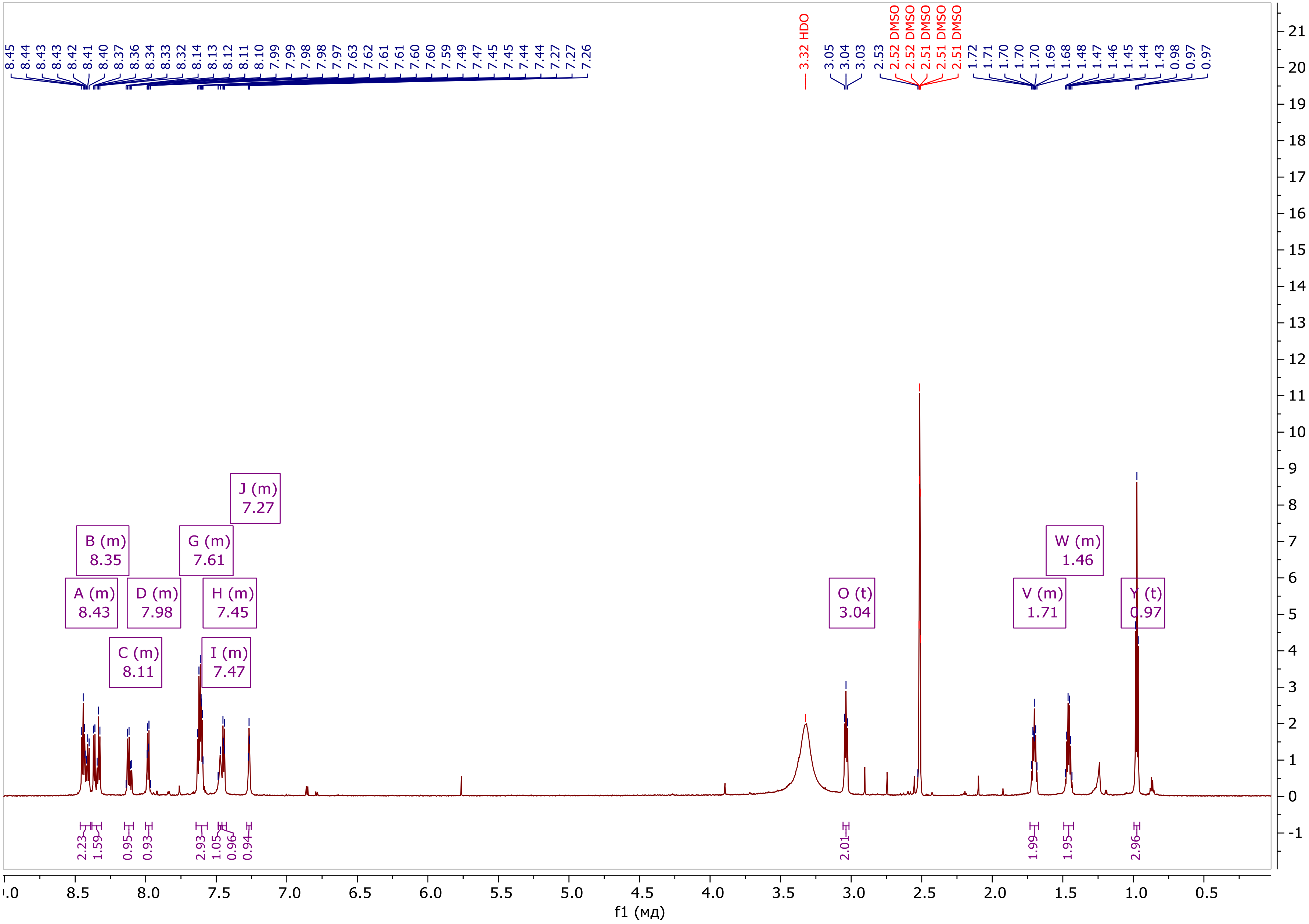

^{13}C NMR spectrum of **15a**

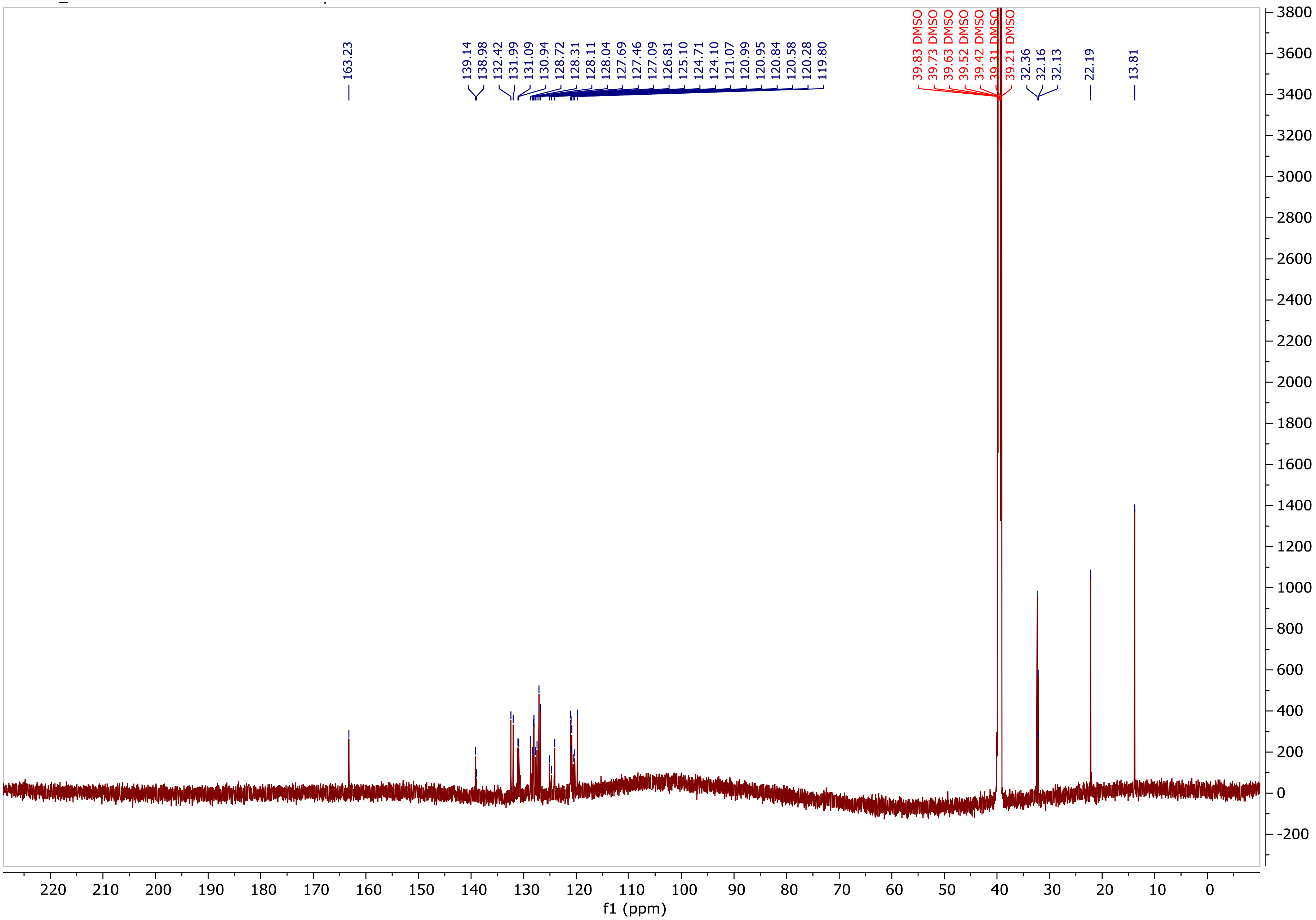

^{1}H NMR spectrum of **9b**

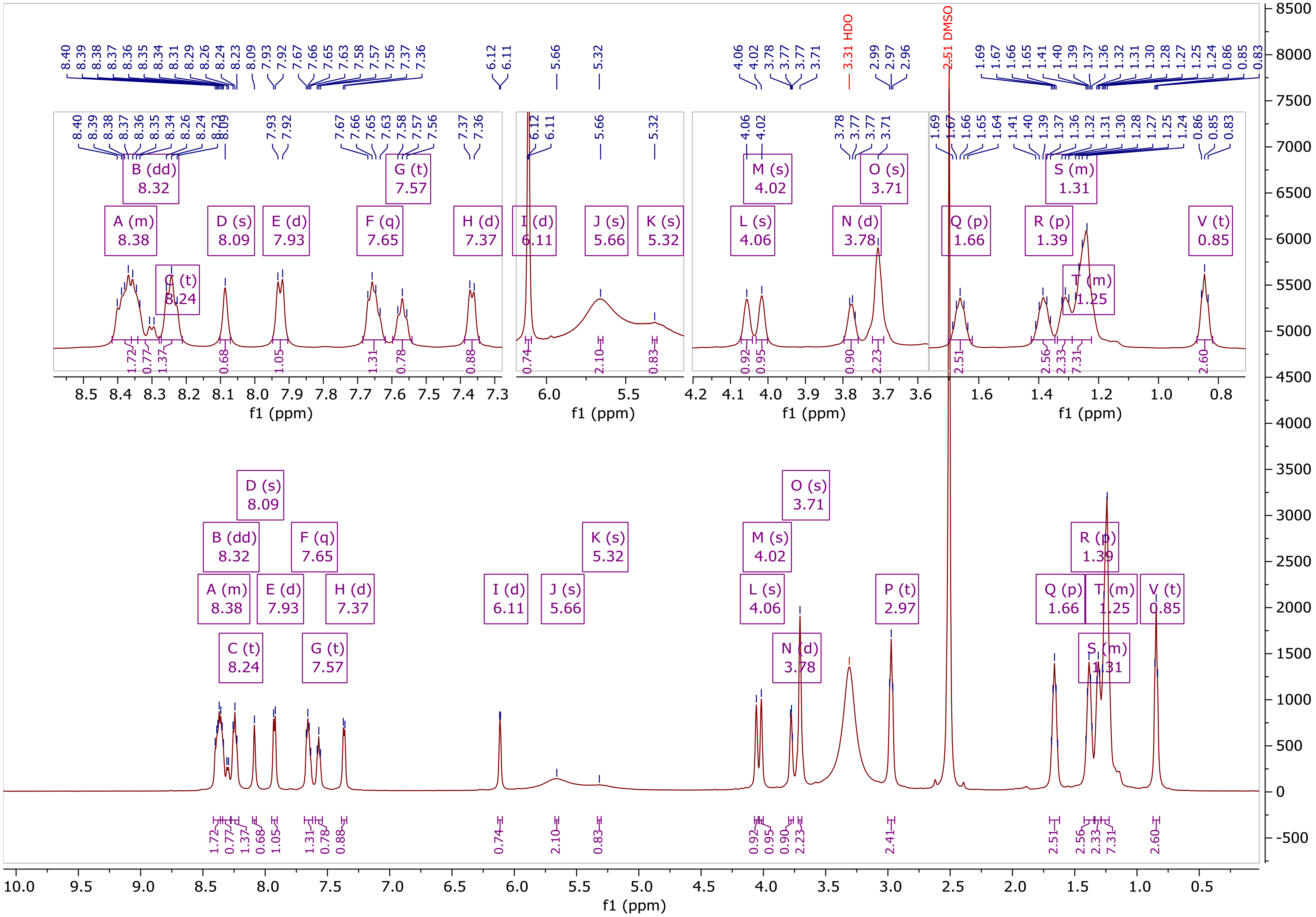

^{13}C NMR spectrum of **9b**

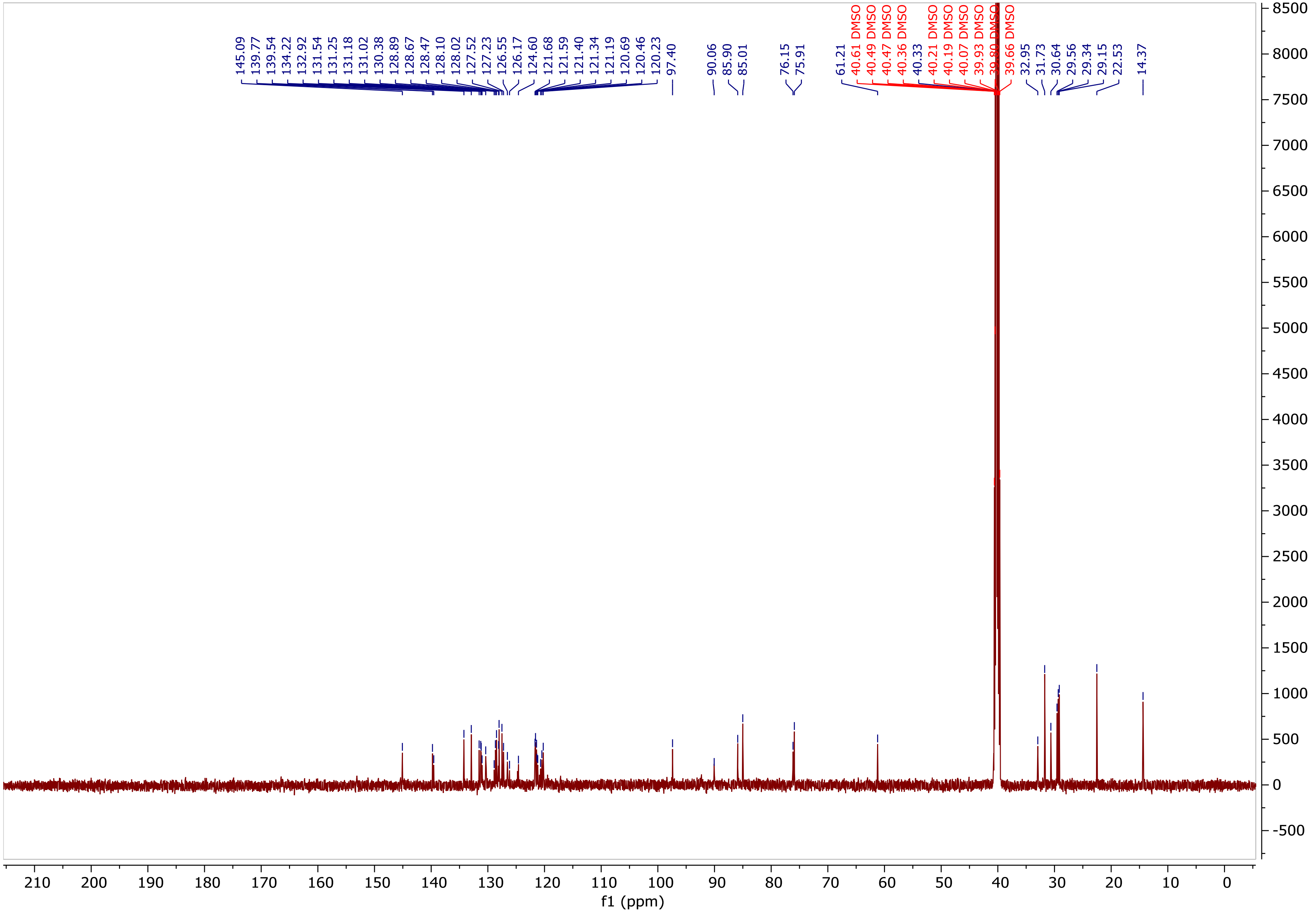

^{1}H NMR spectrum of **10b**

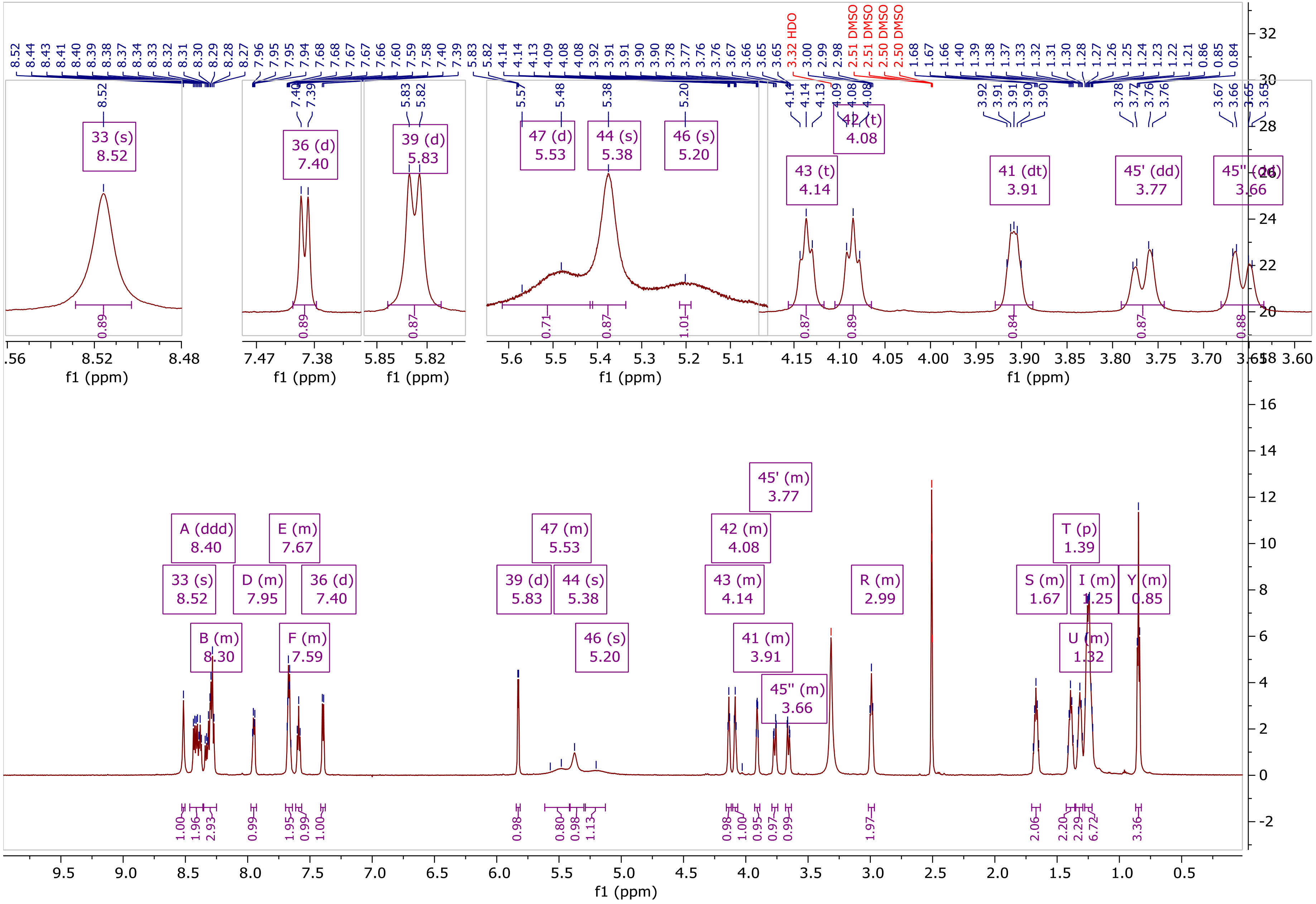

^{13}C NMR spectrum of **10b**

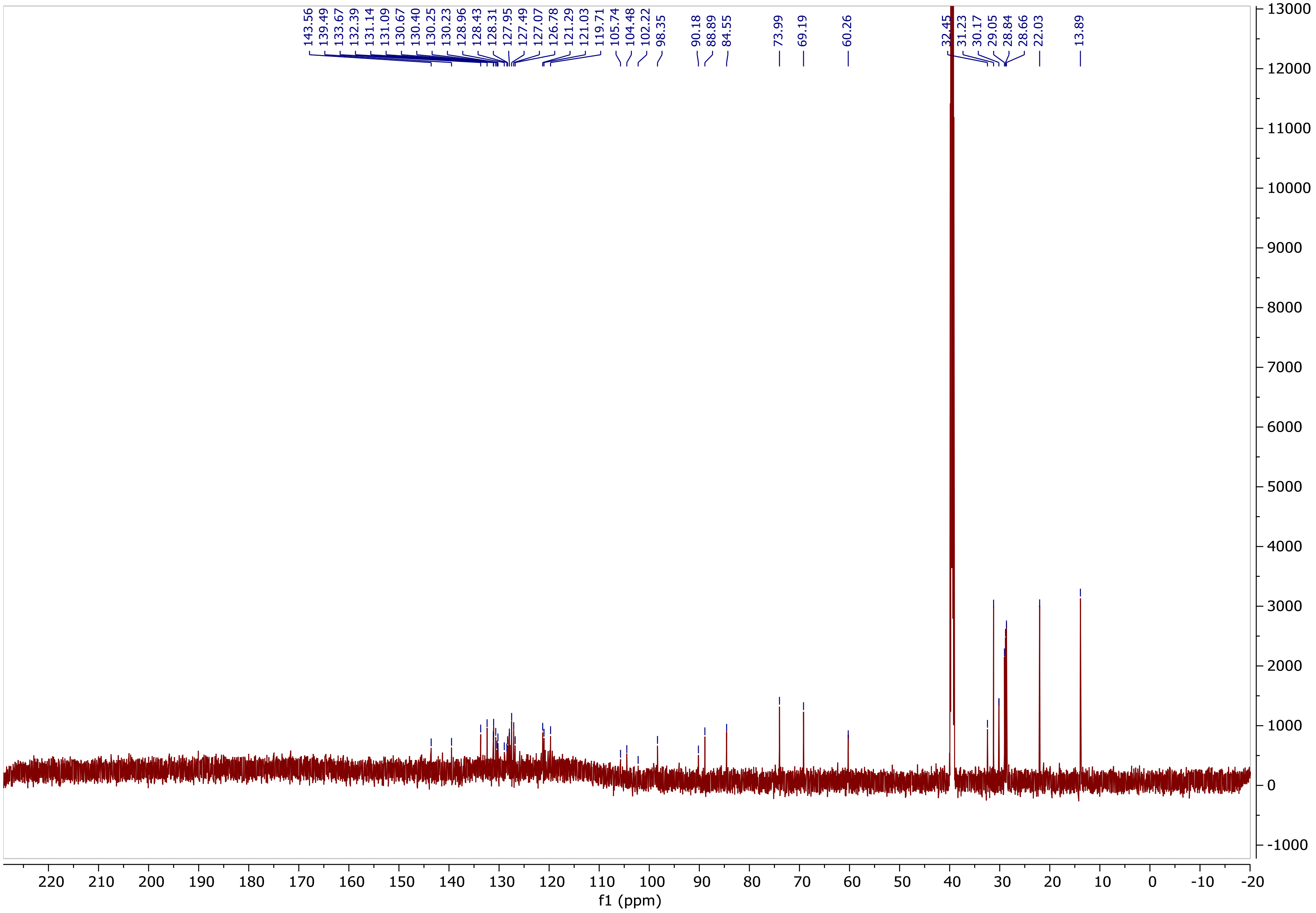

^{1}H NMR spectrum of **11b**

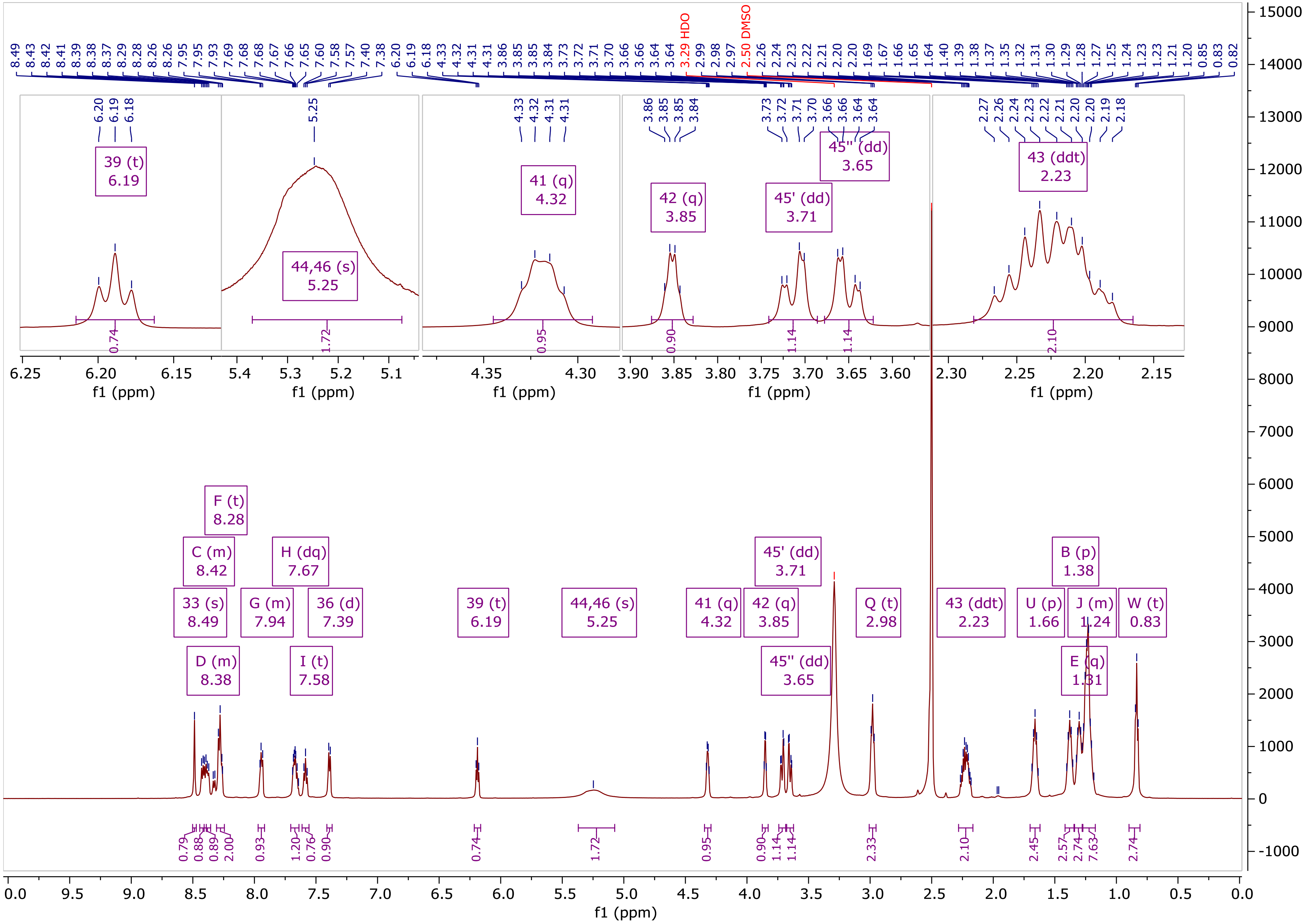

^{13}C NMR spectrum of **11b**

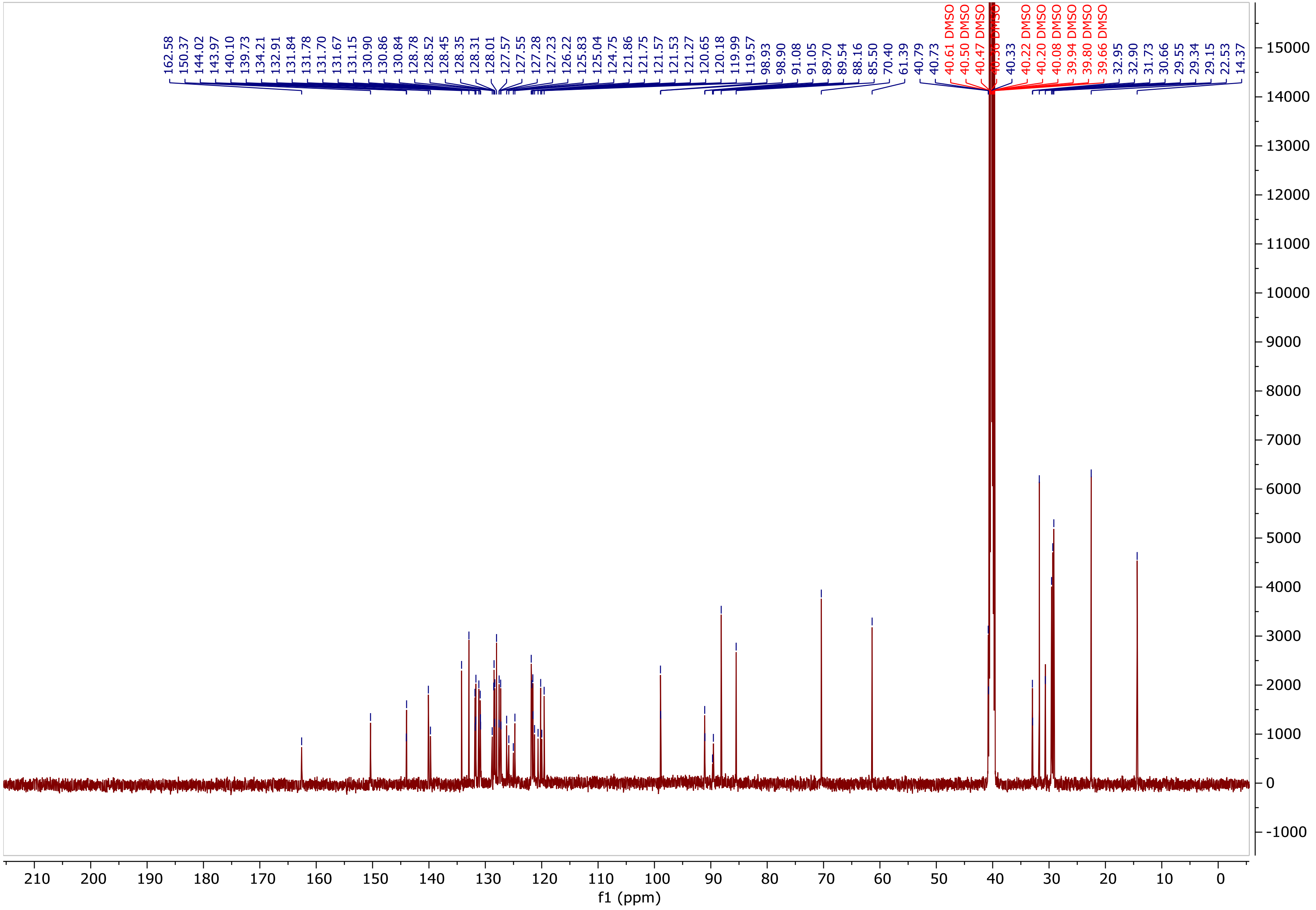

^{1}H NMR spectrum of **12b**

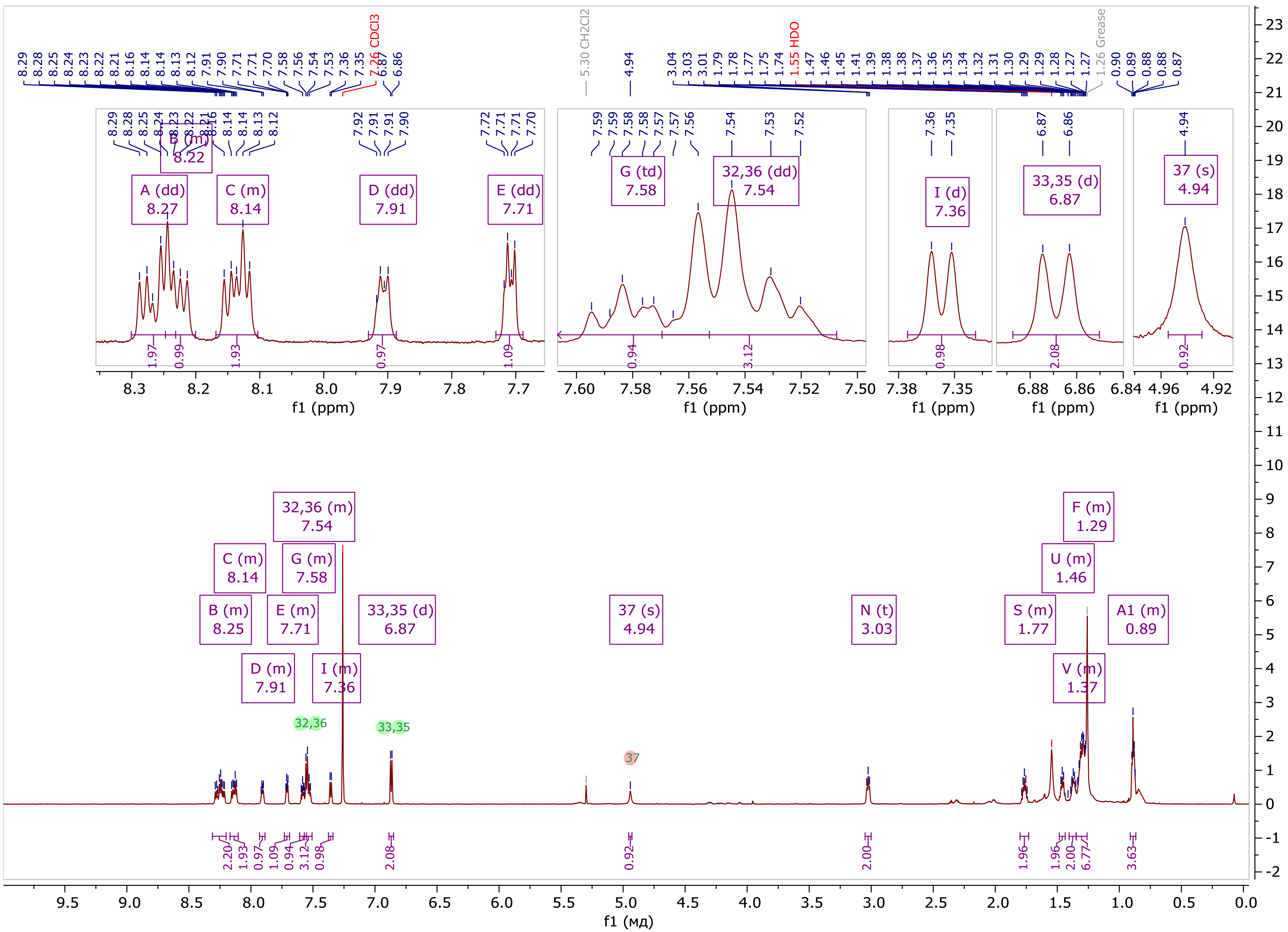

^{13}C NMR spectrum of **12b**

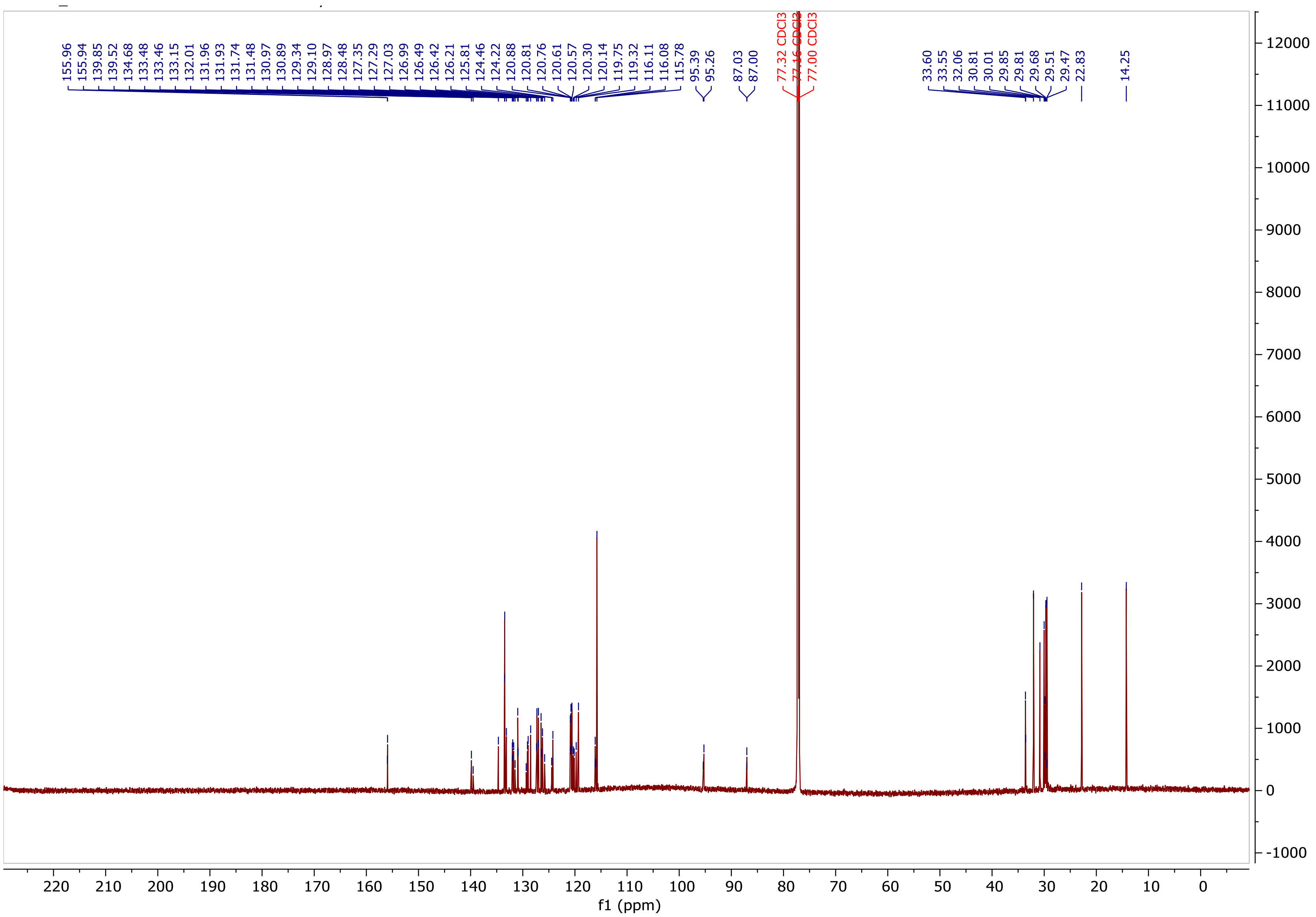

^{1}H NMR spectrum of **13b**

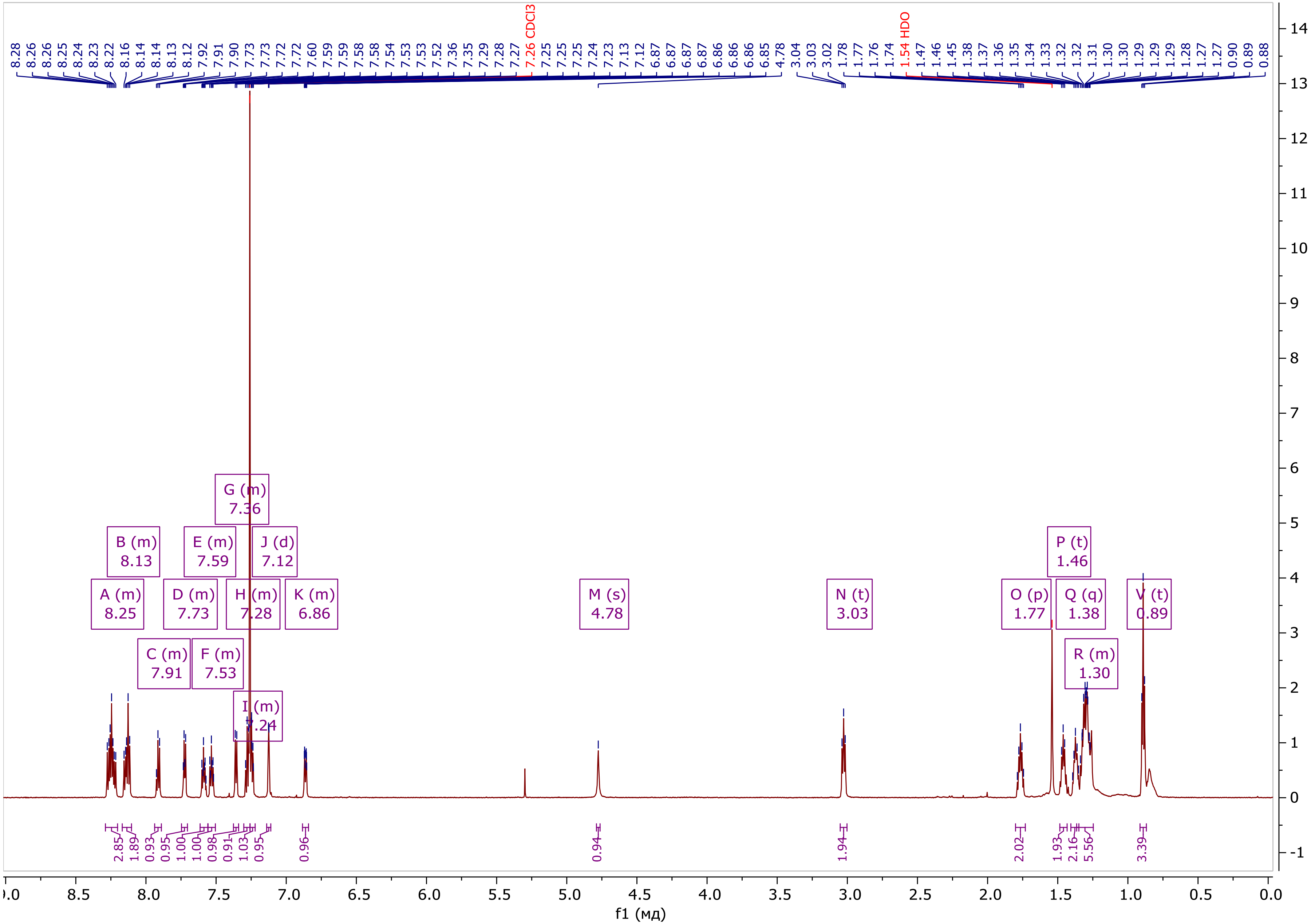

^{13}C NMR spectrum of **13b**

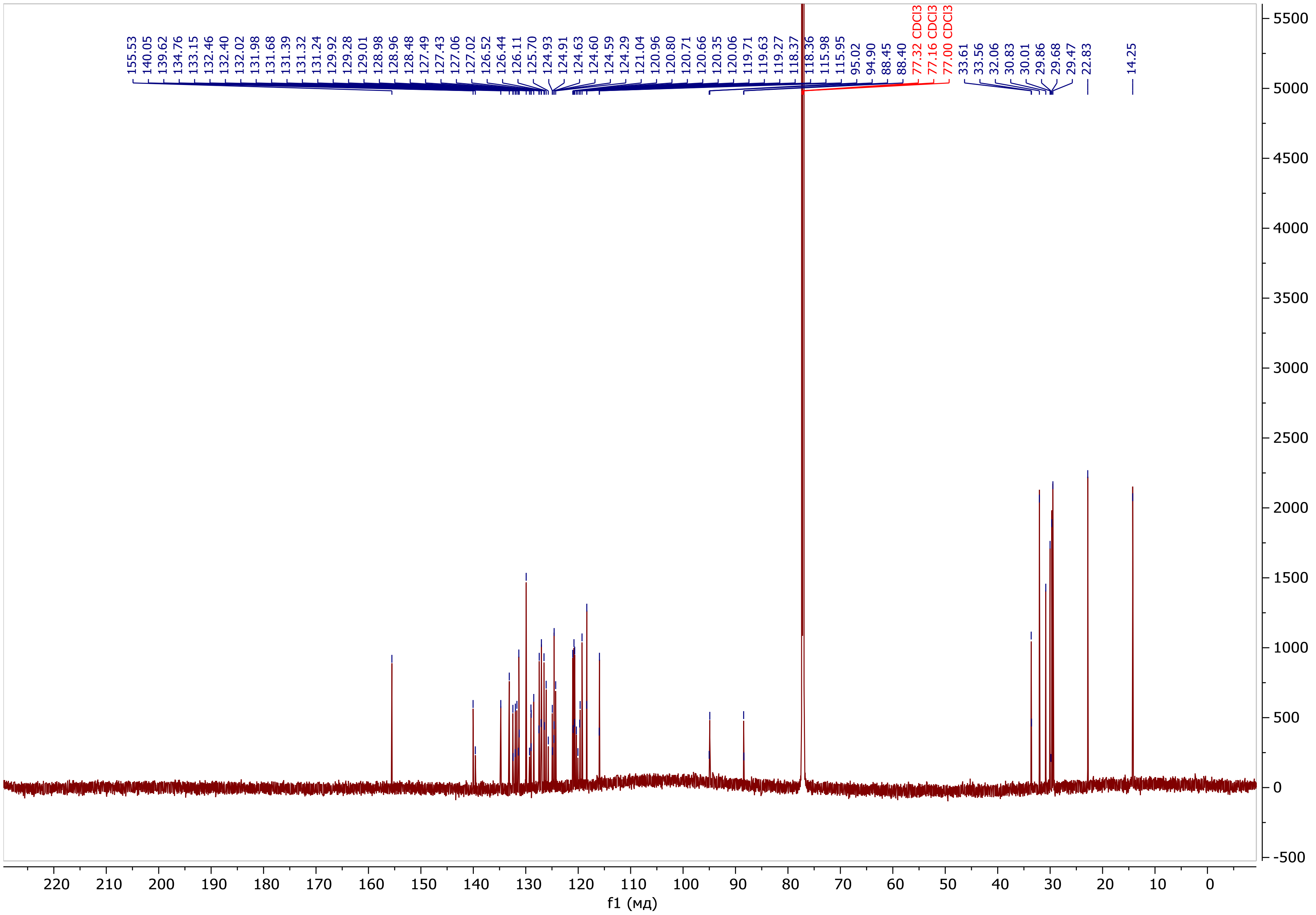

^{1}H NMR spectrum of **14b**

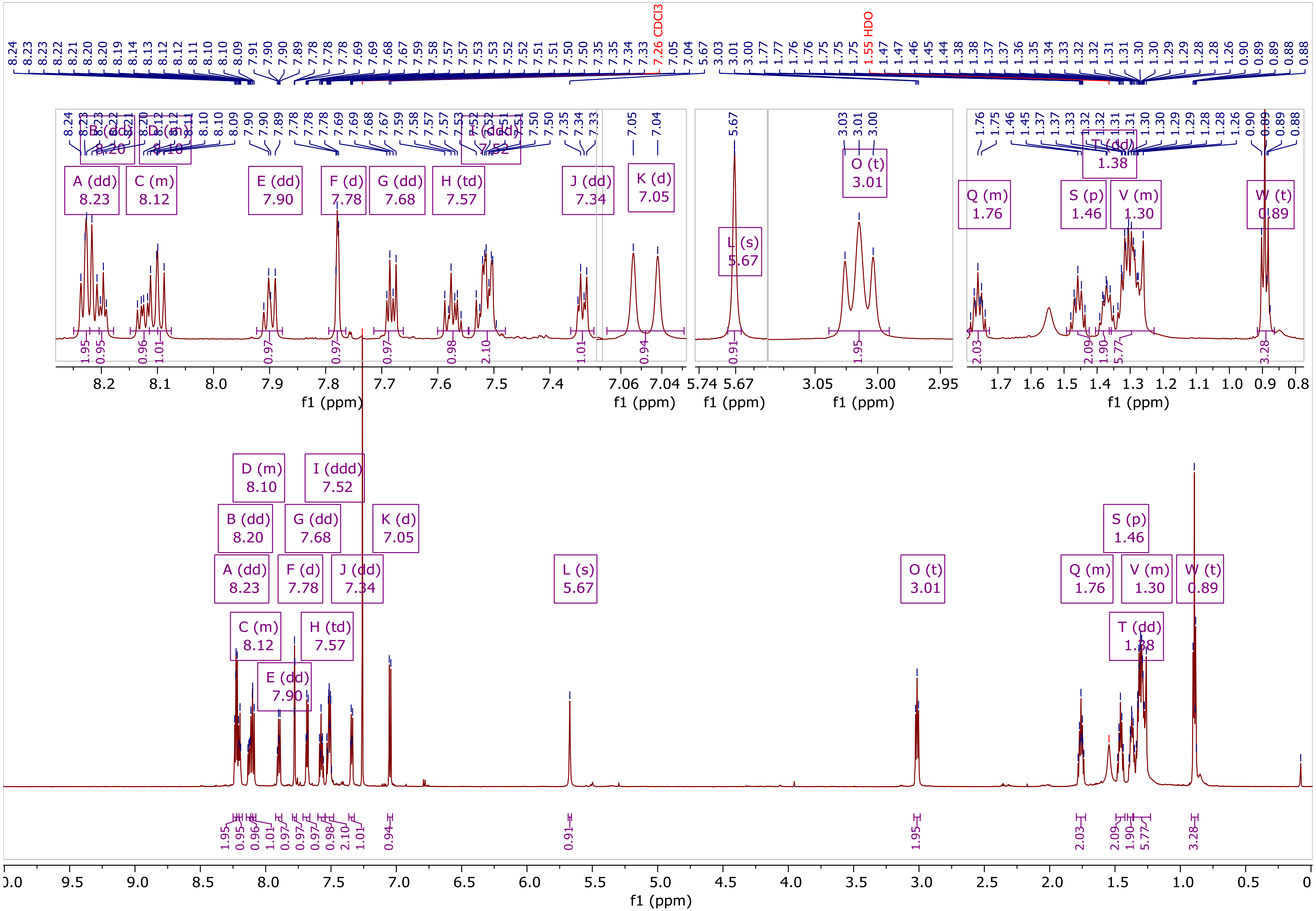

^{13}C NMR spectrum of **14b**

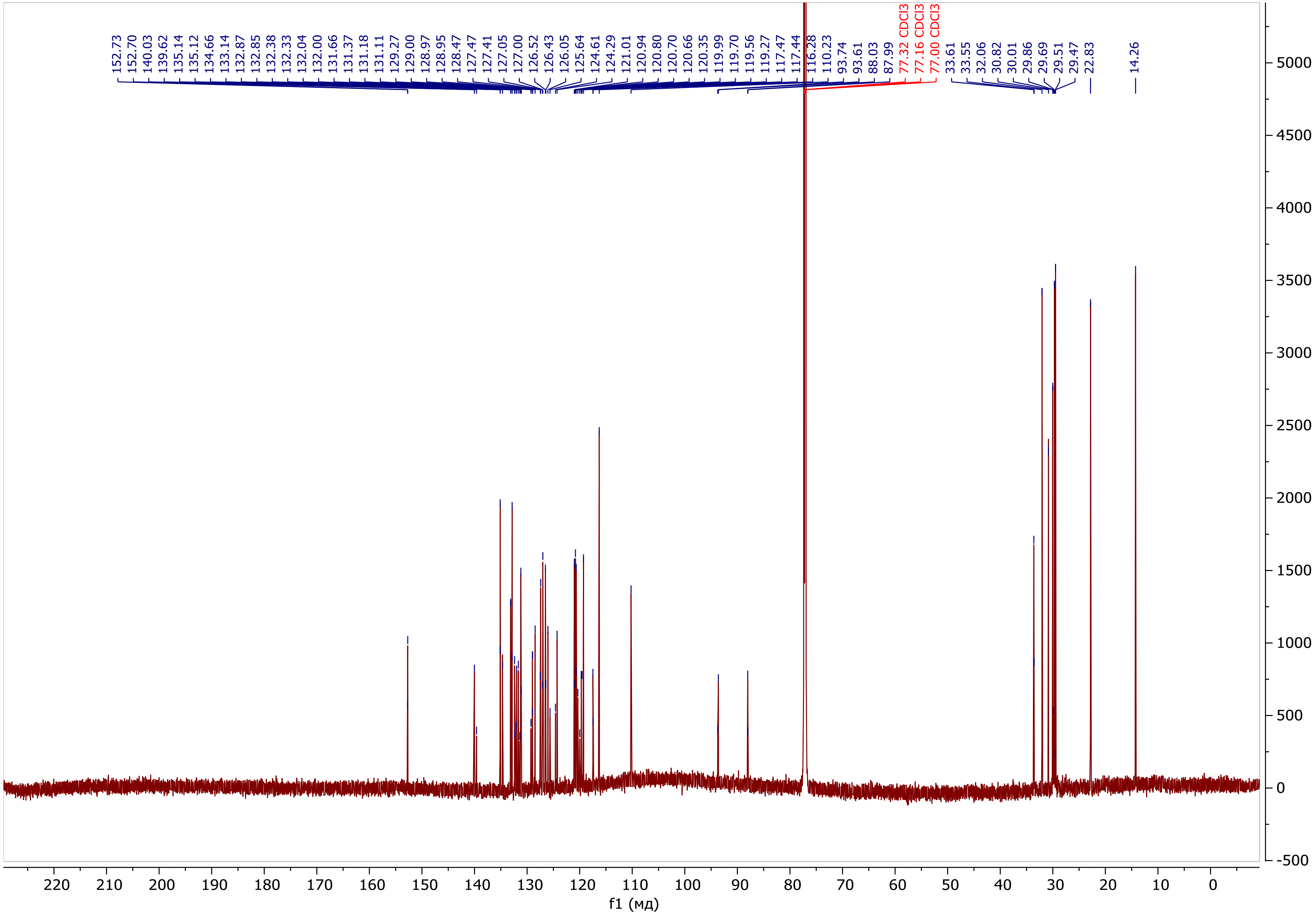

^{1}H NMR spectrum of **15b**

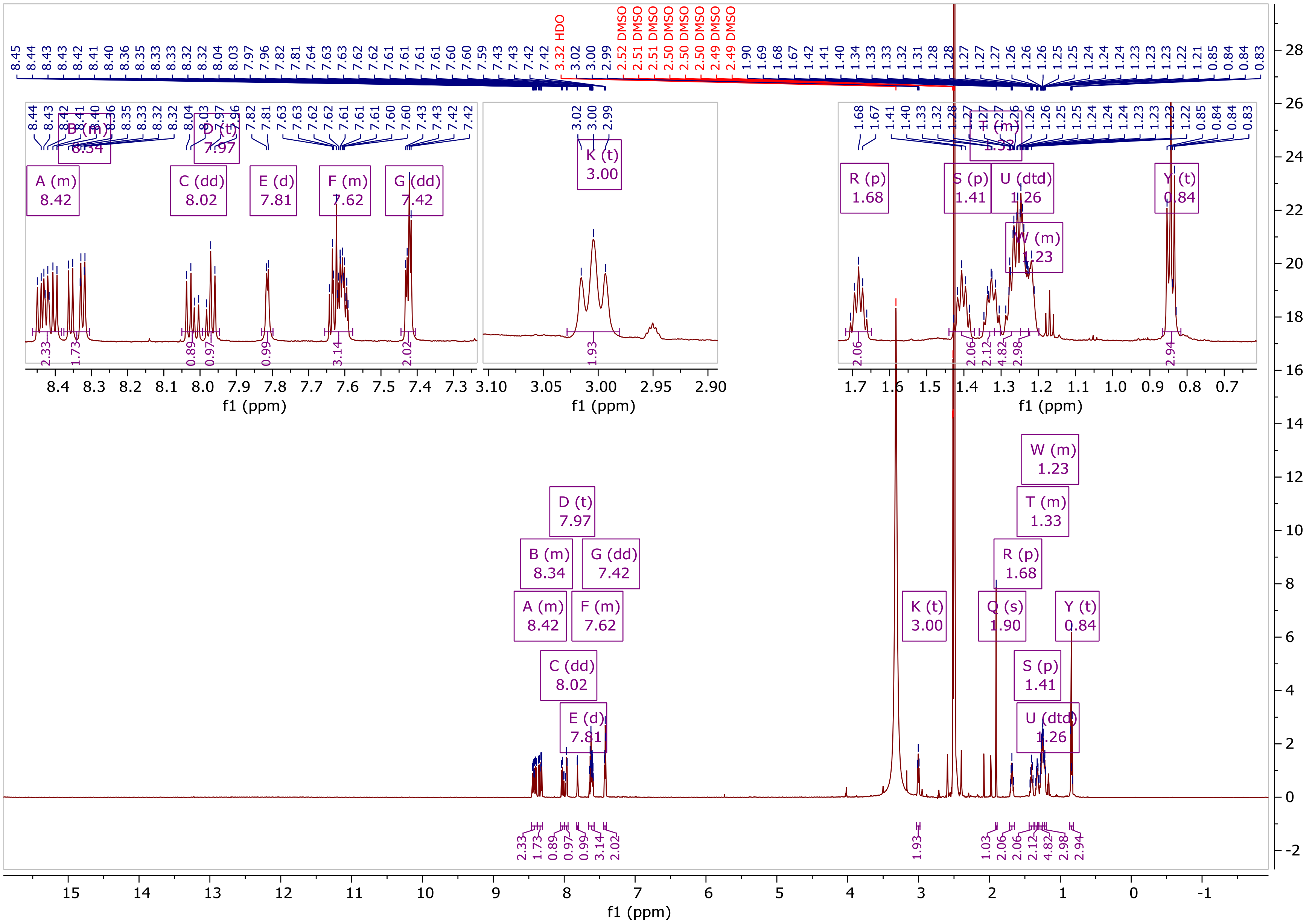

^{13}C NMR spectrum of **15b**

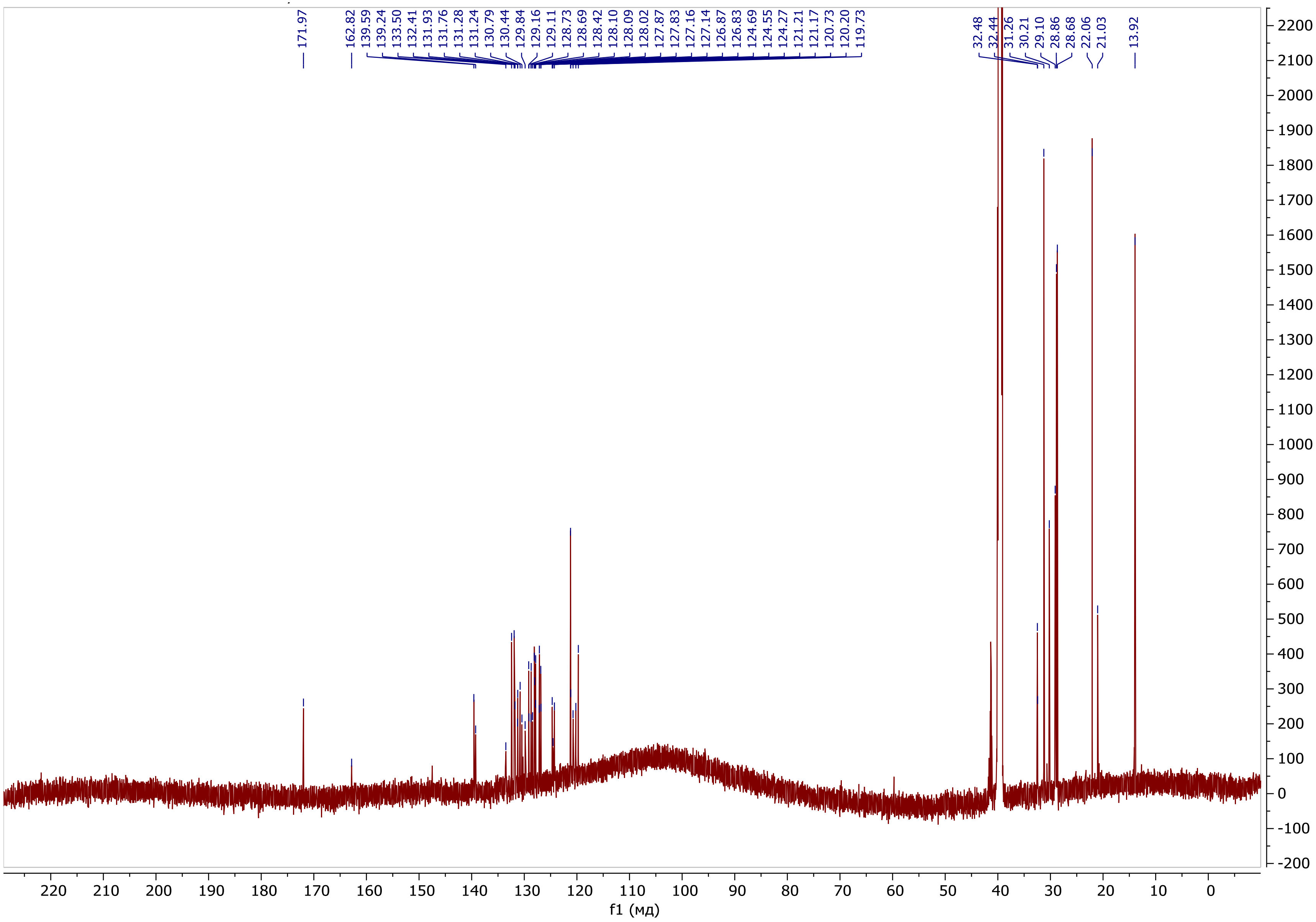

^{1}H NMR spectrum of **9c**

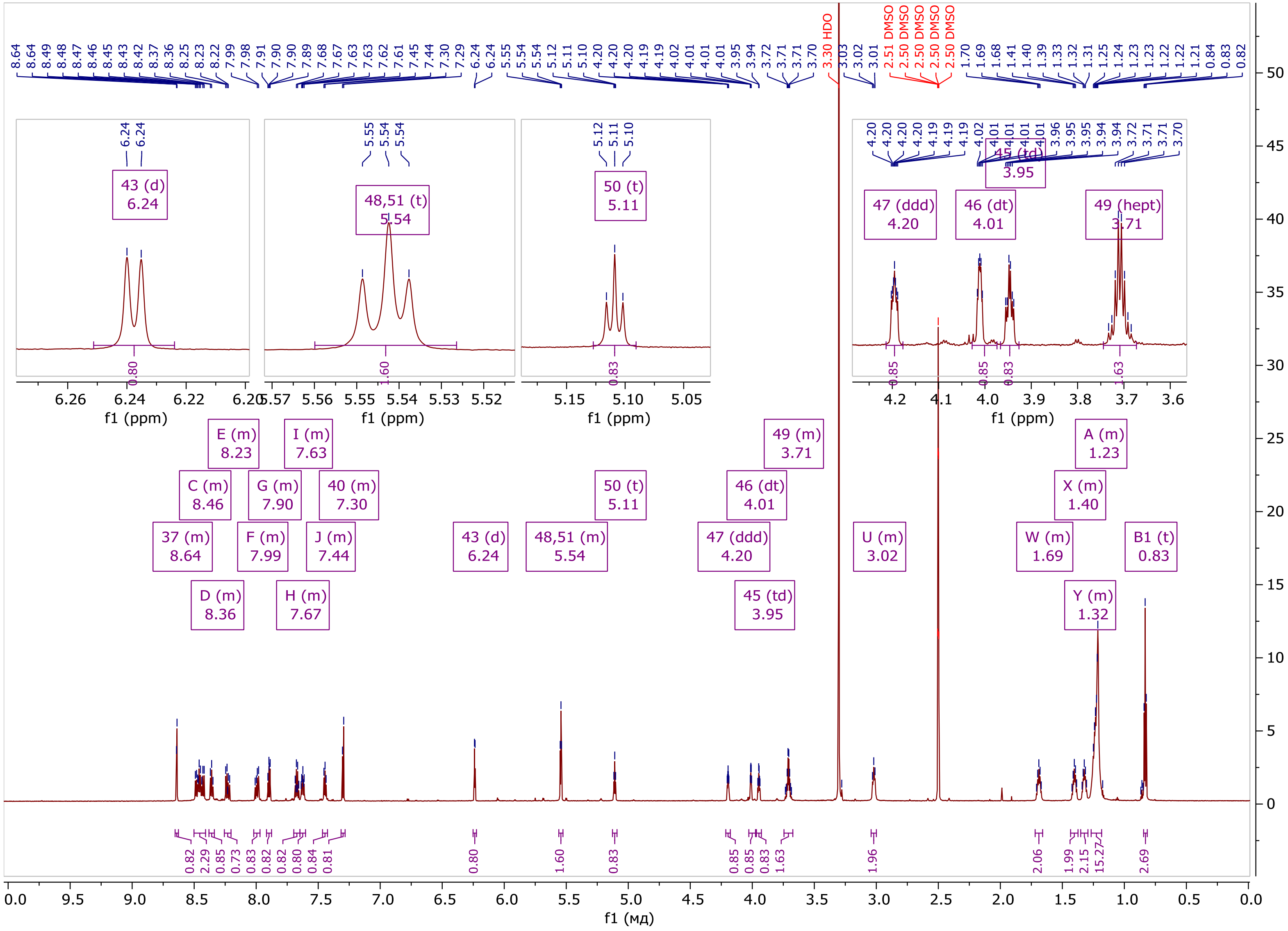

^{13}C NMR spectrum of **9c**

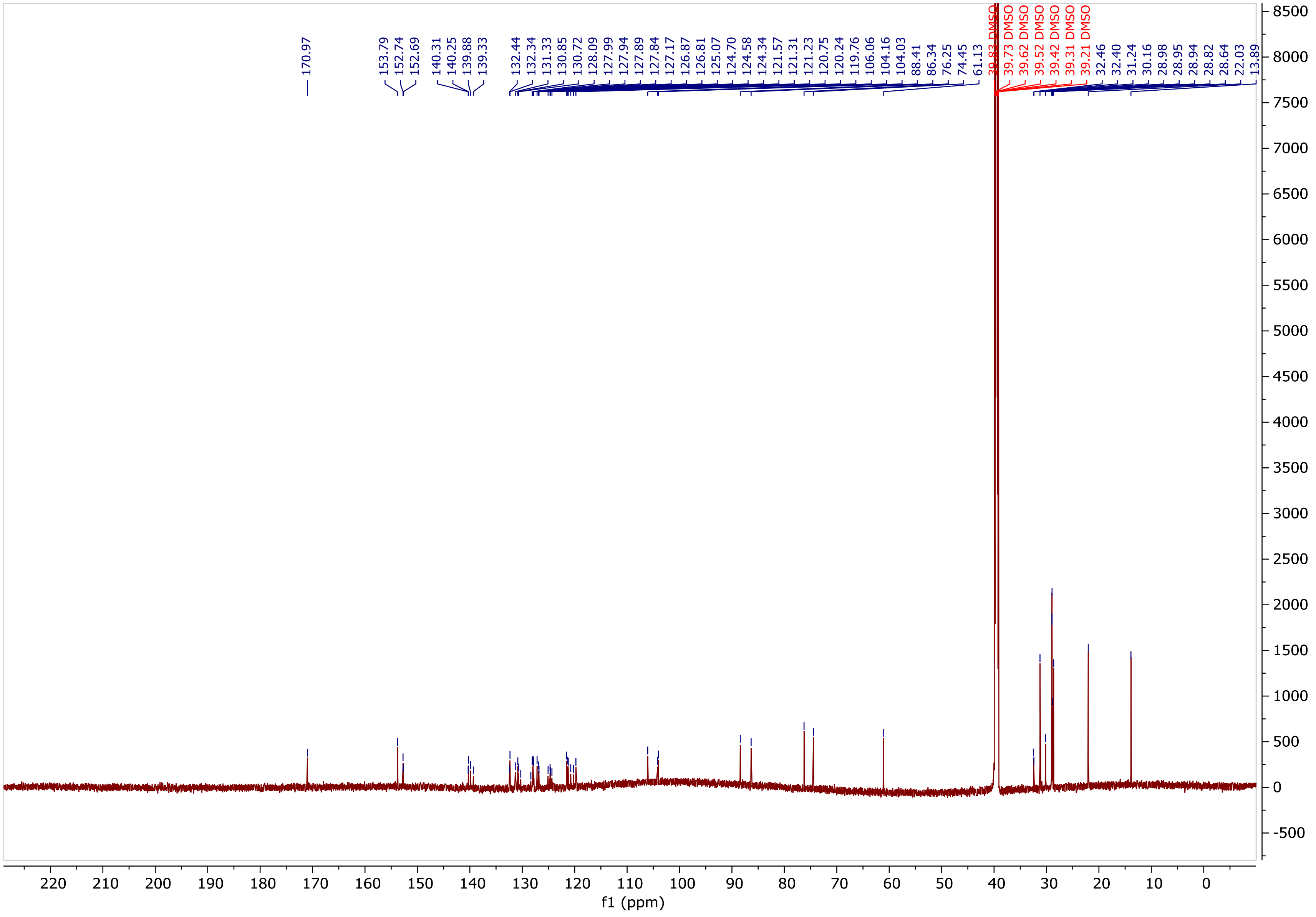

^{1}H NMR spectrum of **10c**

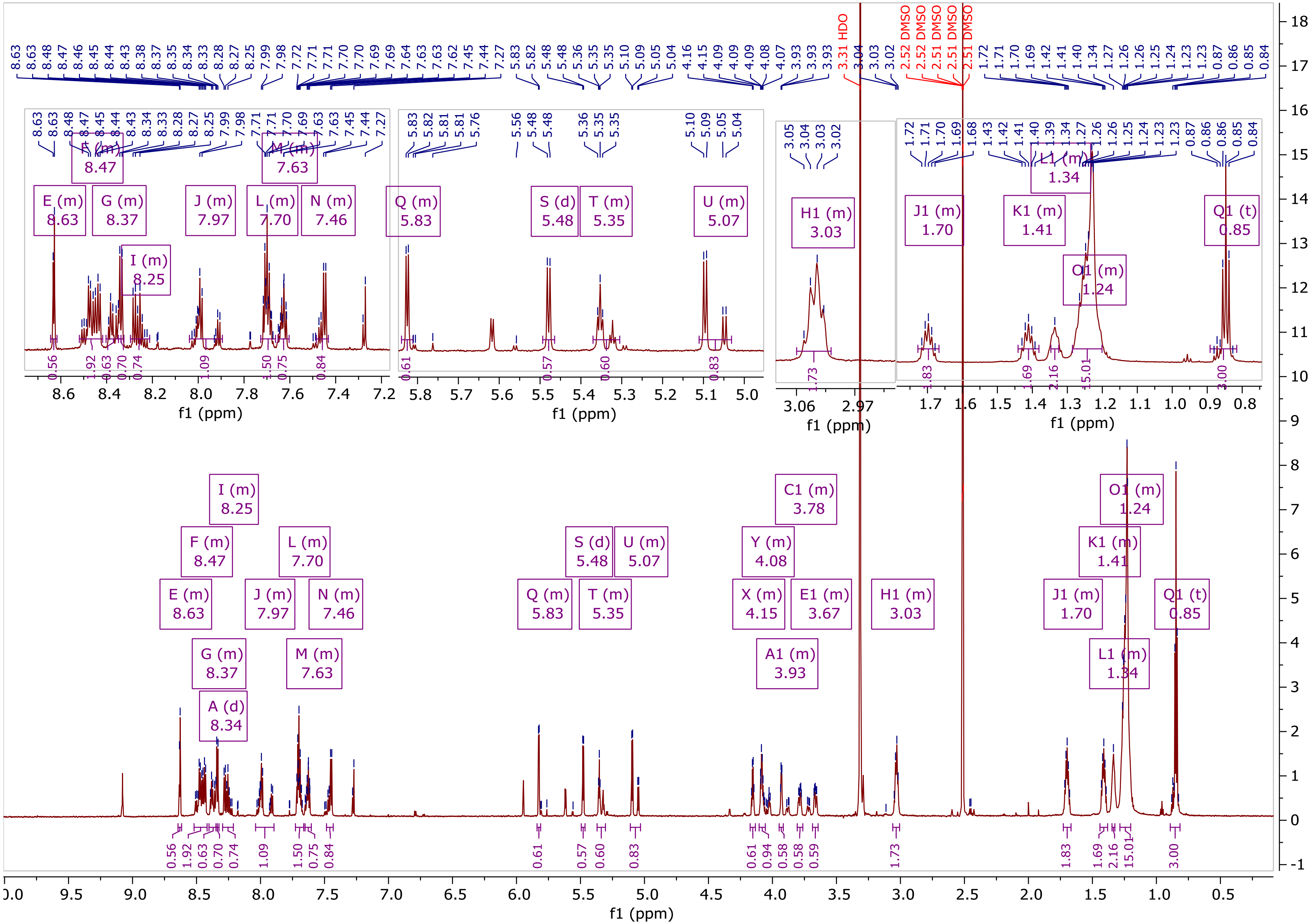

^{13}C NMR spectrum of **10c**

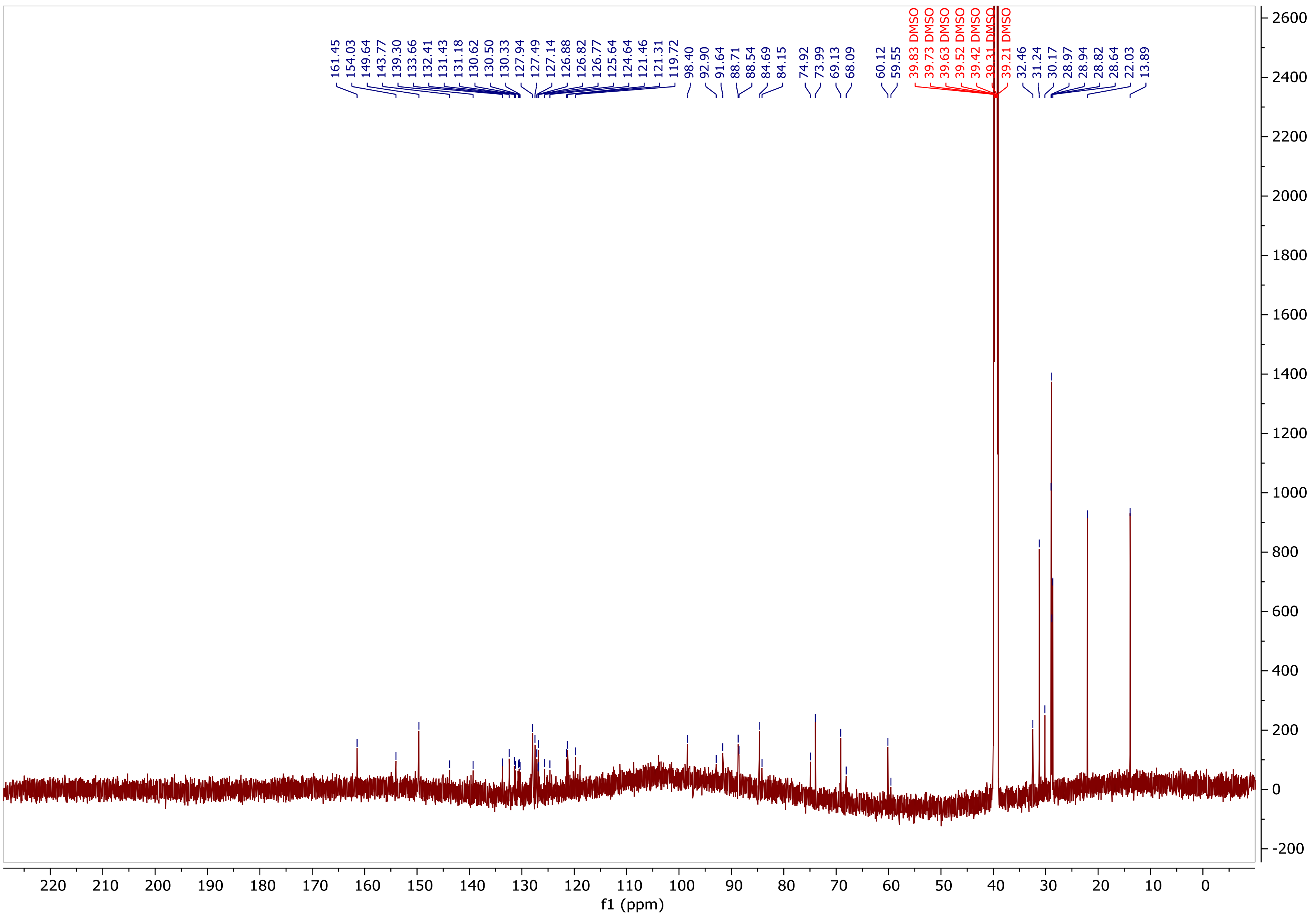

^{1}H NMR spectrum of **11c**

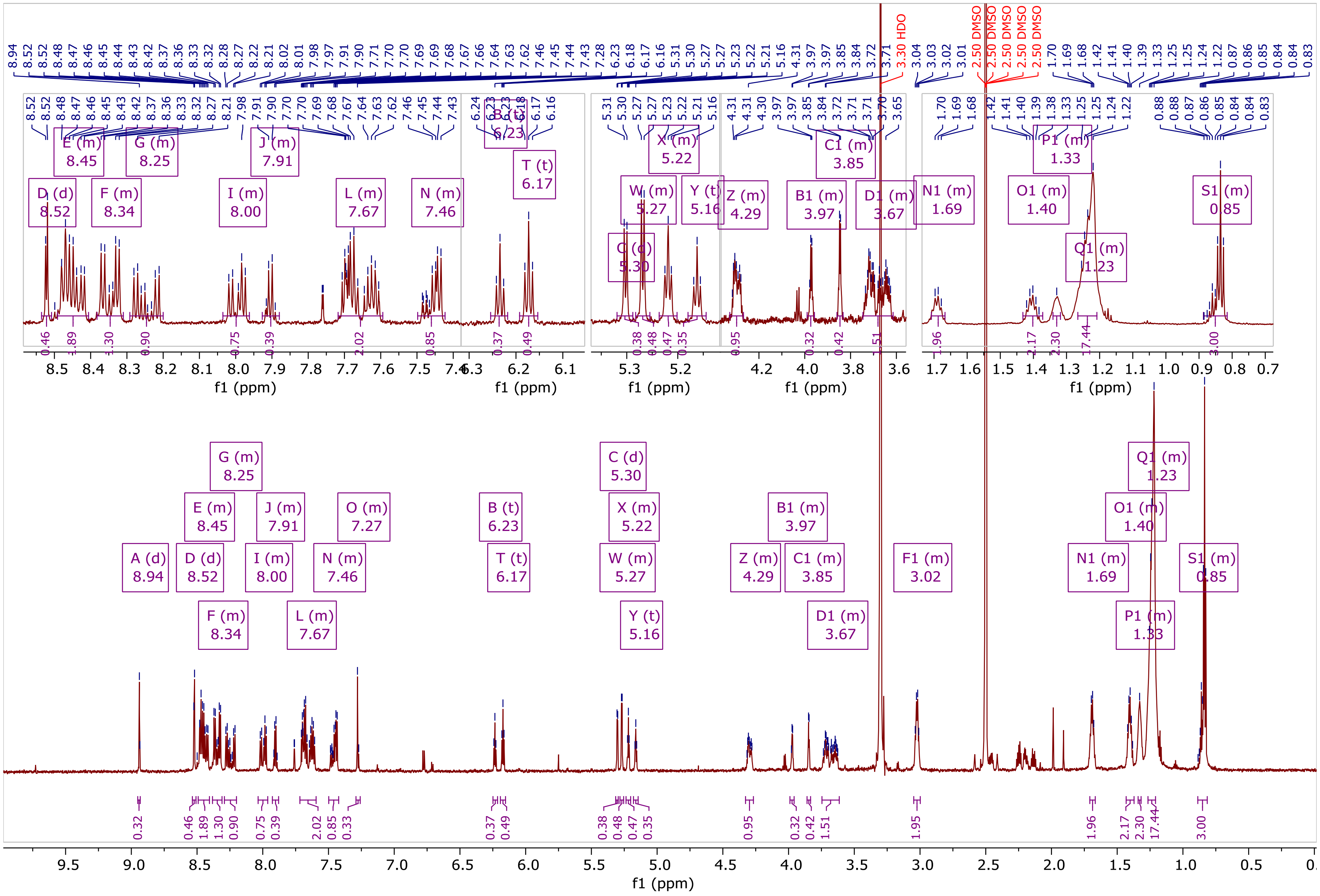

^{13}C NMR spectrum of **11c**

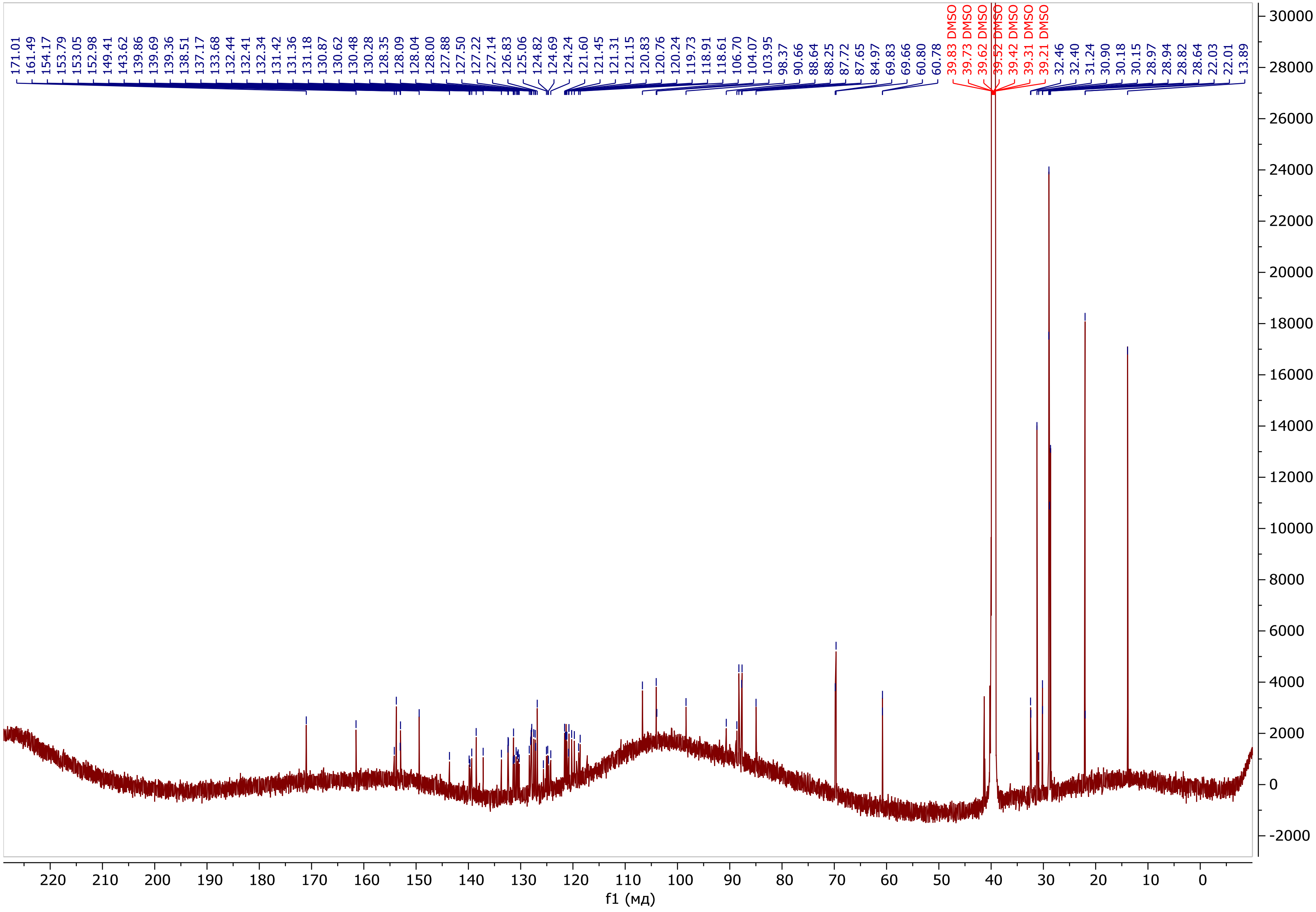

^{1}H NMR spectrum of **12c**

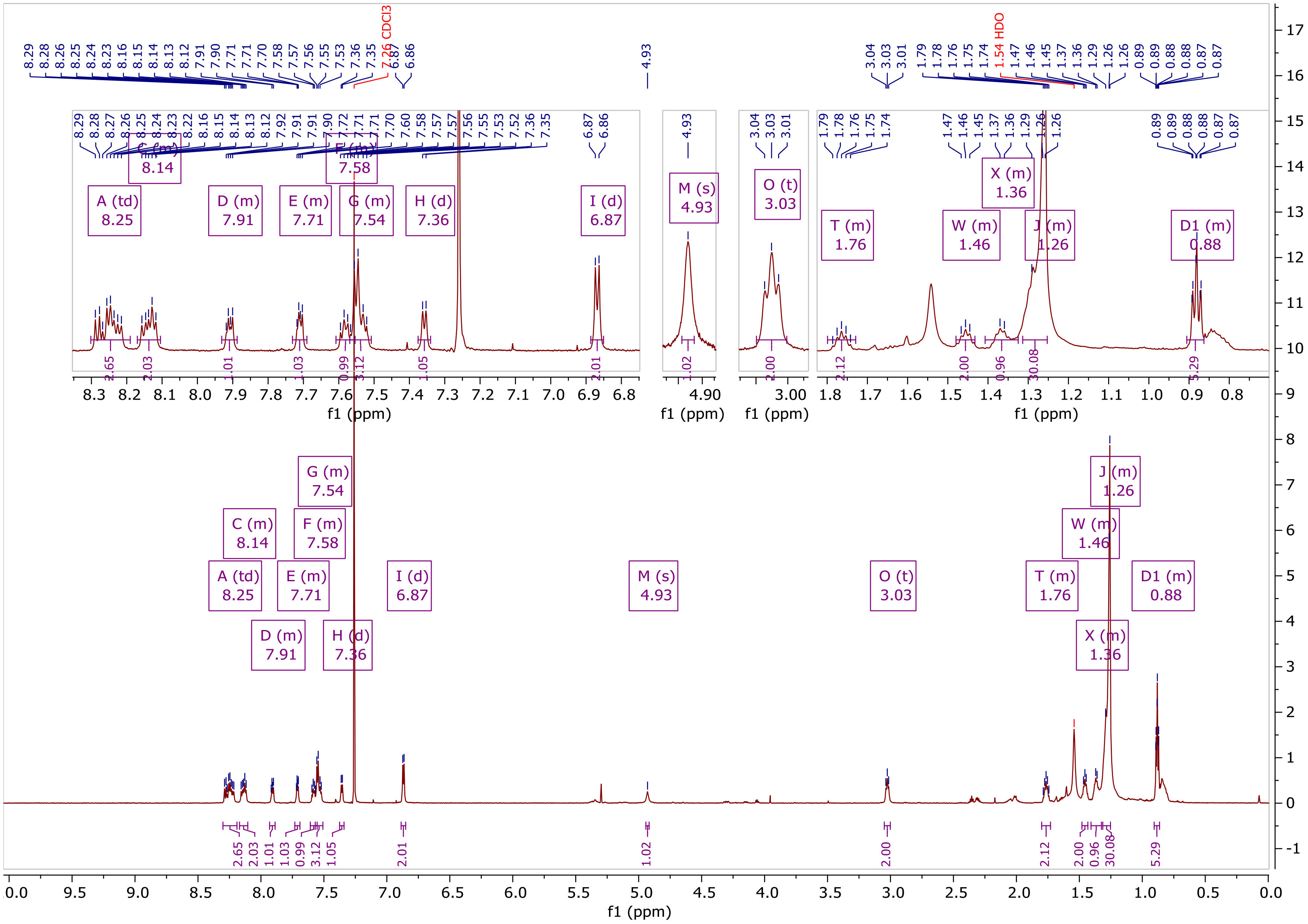

^{13}C NMR spectrum of **12c**

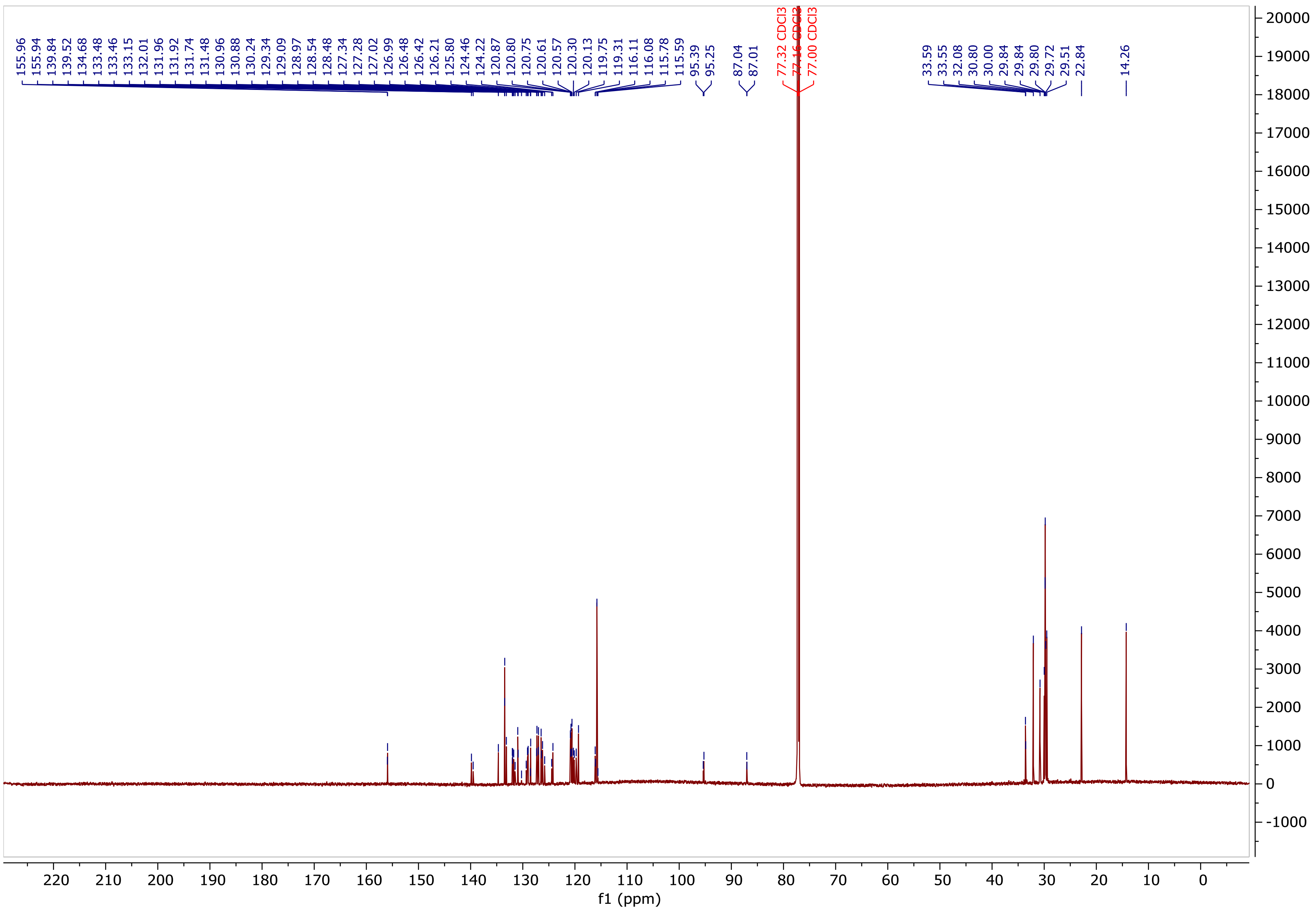

^{1}H NMR spectrum of **13c**

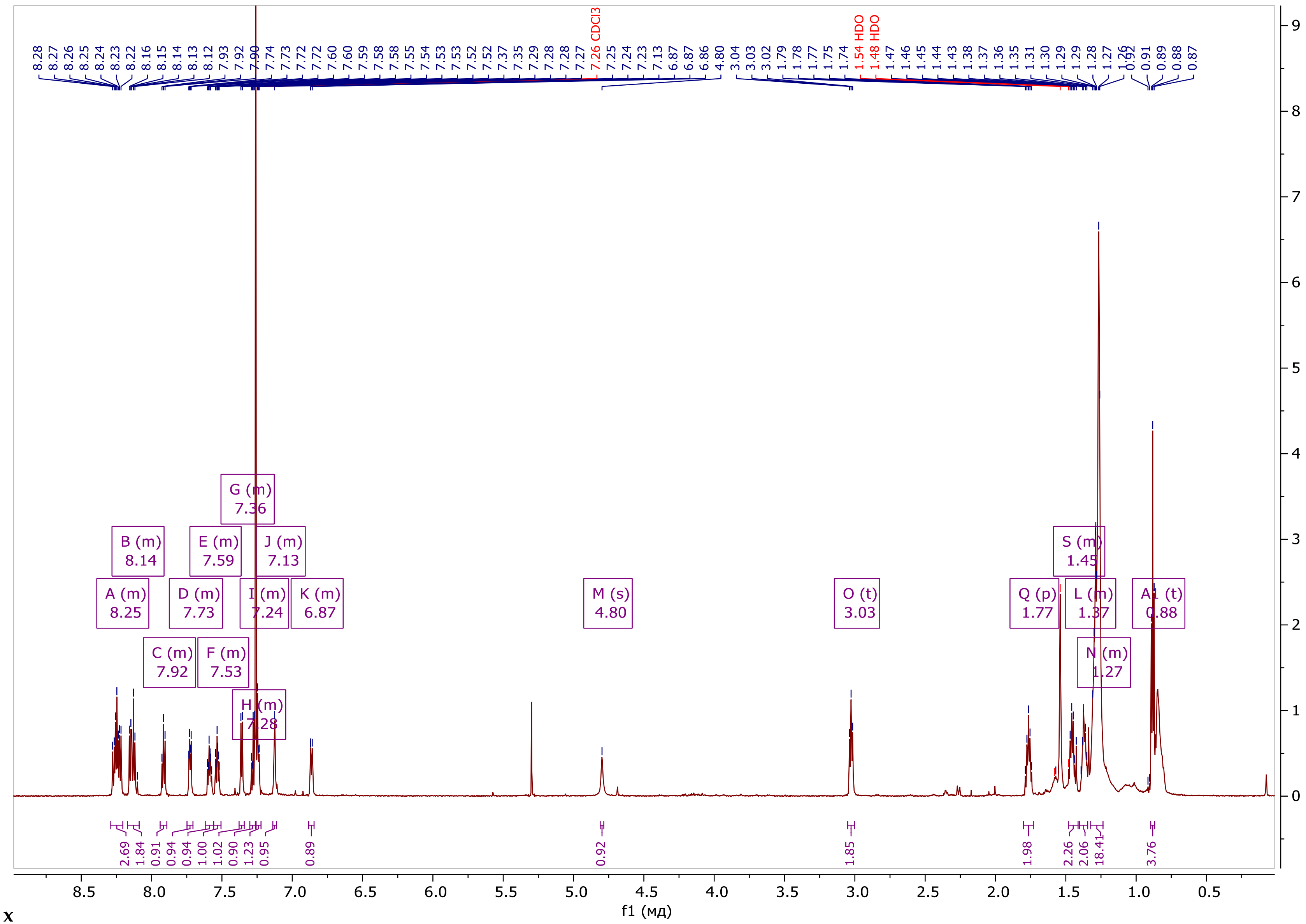

x

^{13}C NMR spectrum of **13c**

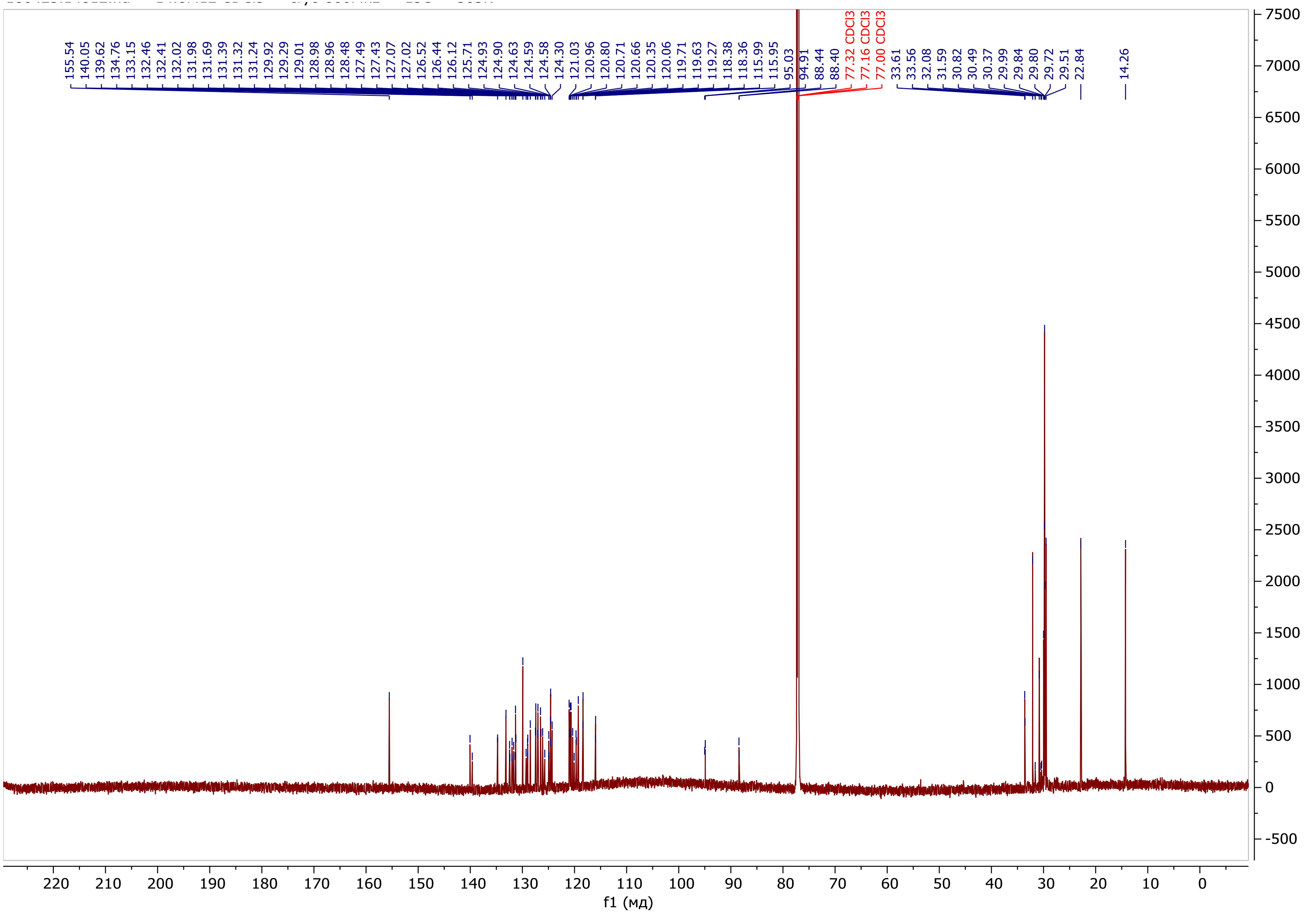

^{1}H NMR spectrum of **14c**

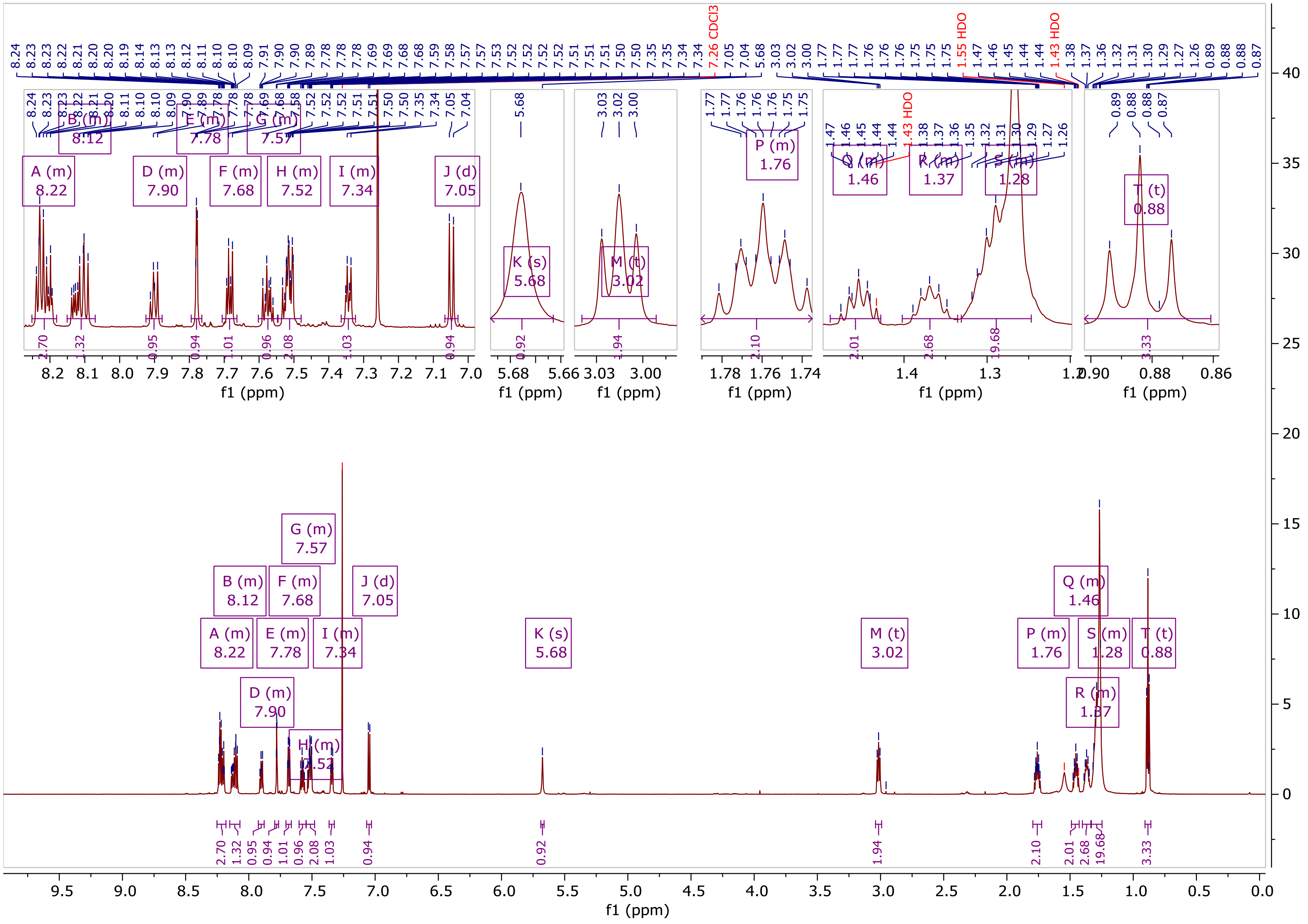

^{13}C NMR spectrum of **14c**

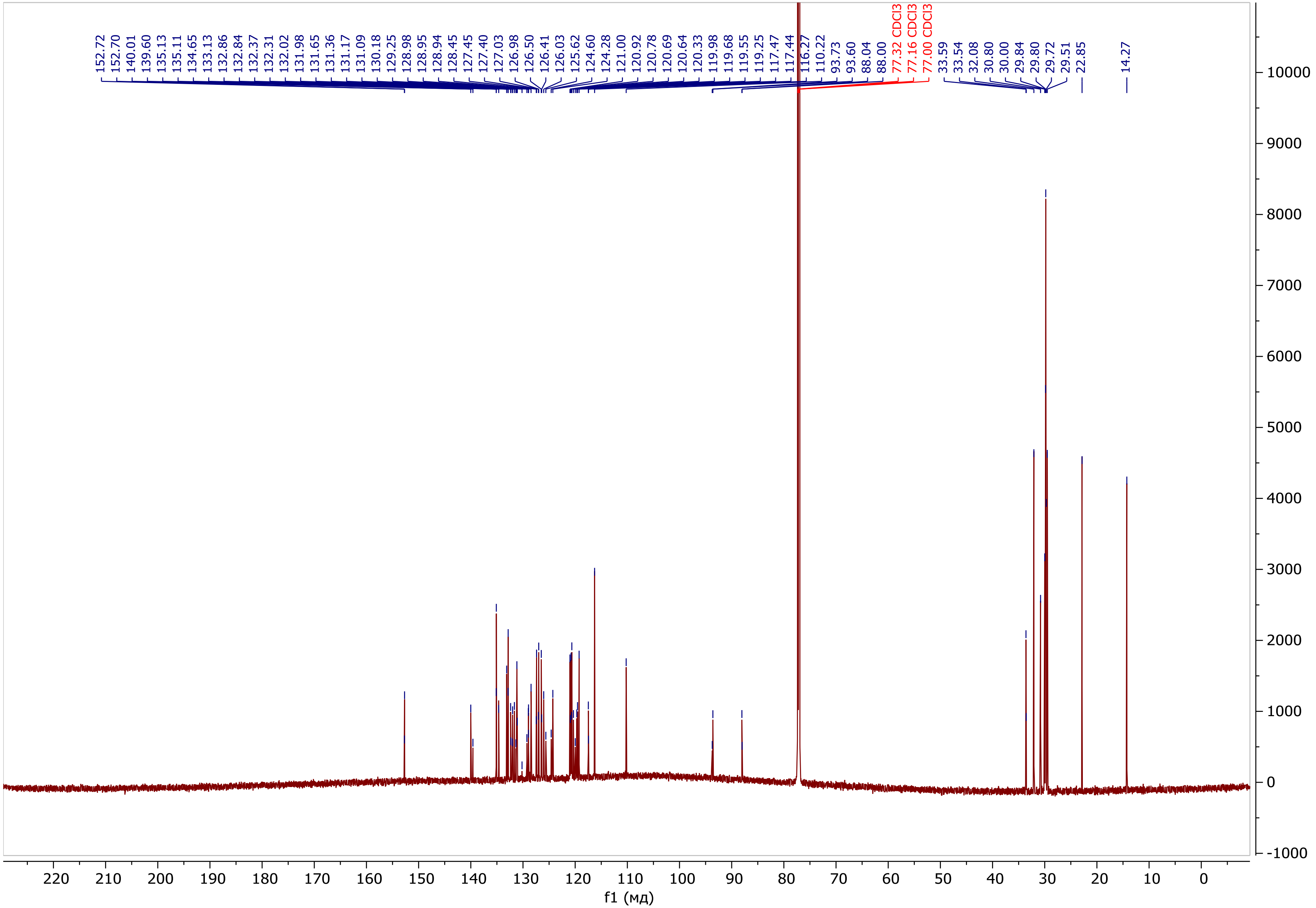

^{1}H NMR spectrum of **15c**

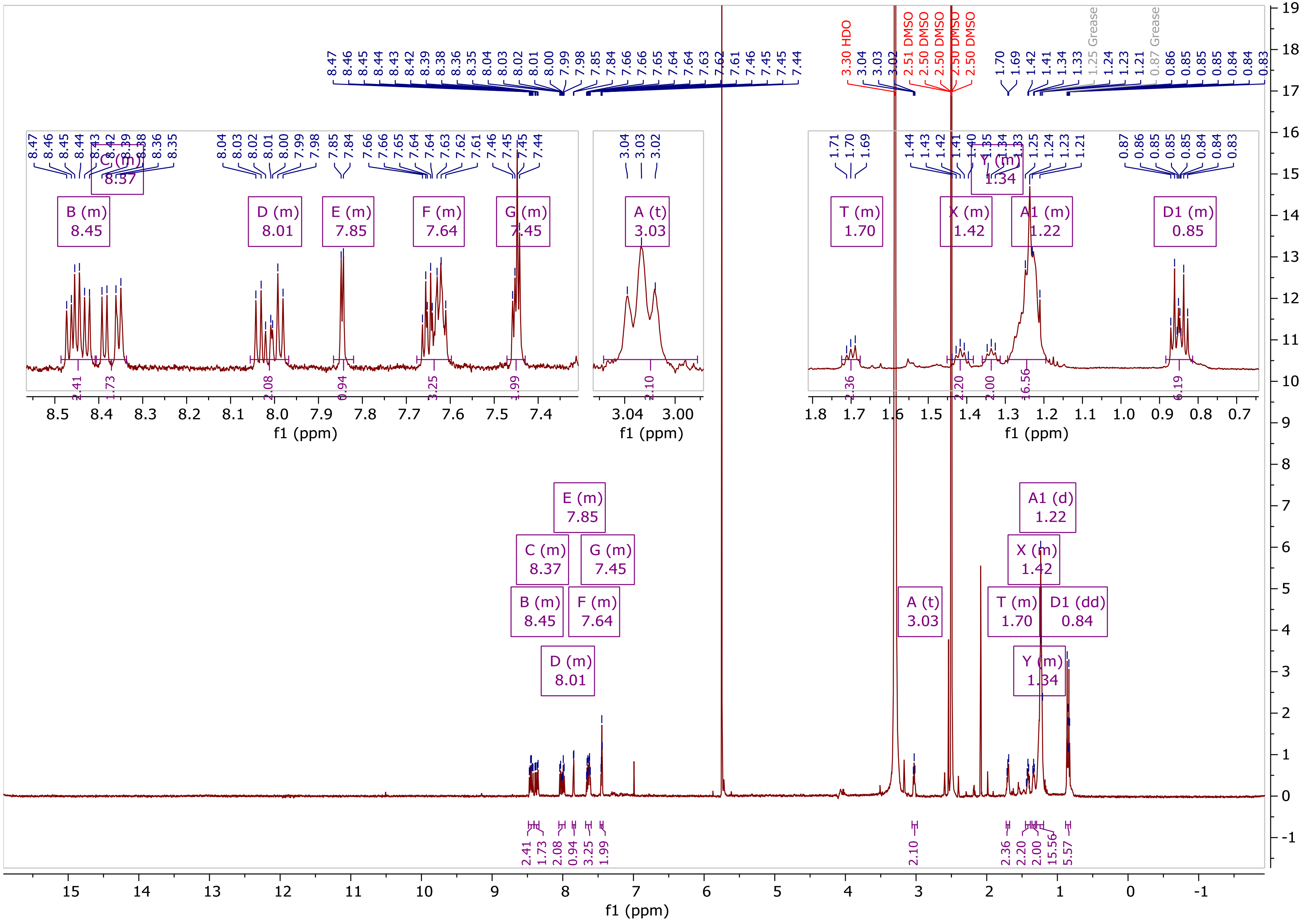

HMBC and HSQC NMR spectra of **15c**

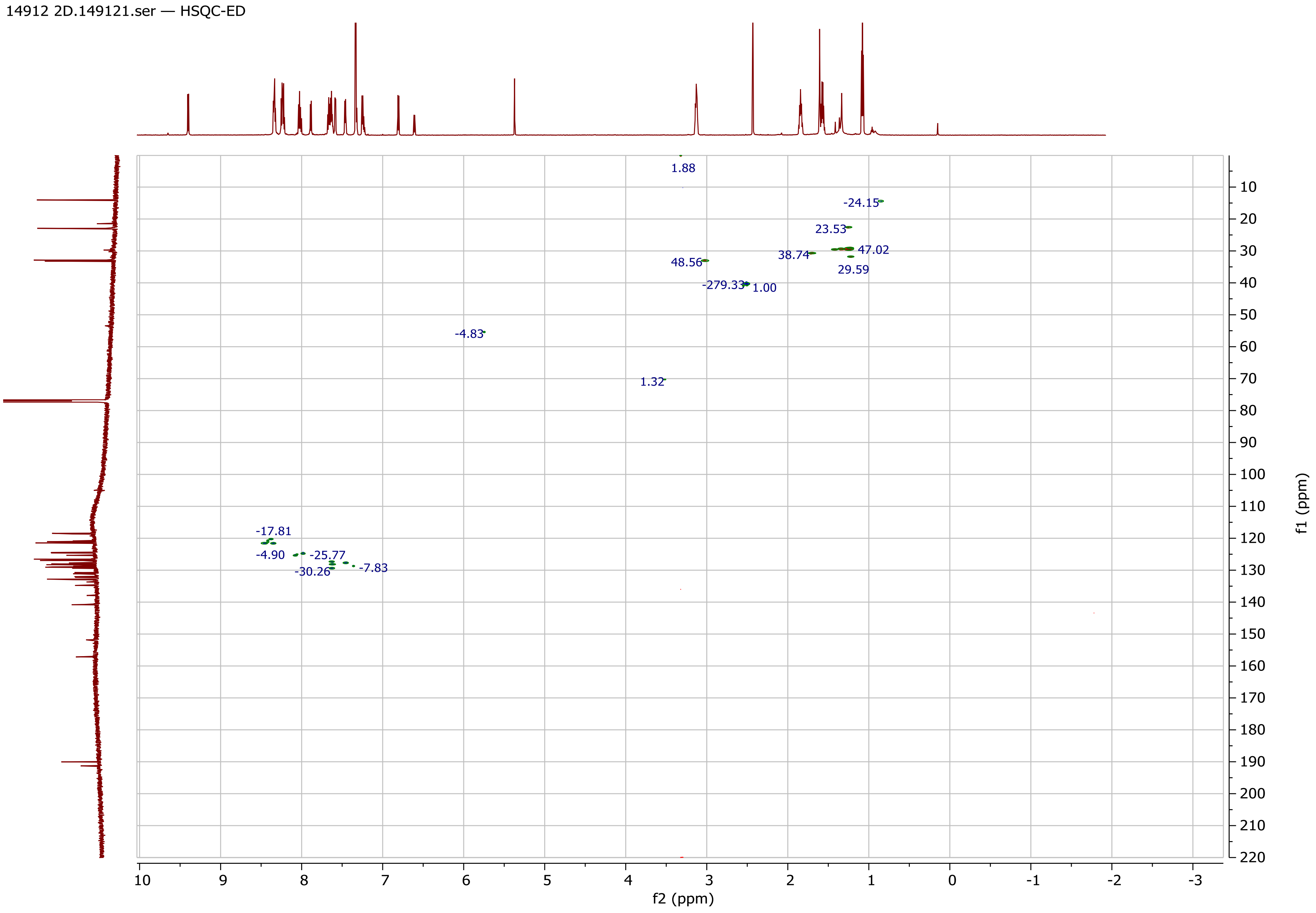

14912 2D.149123.ser — 14.9T12 — HMBC

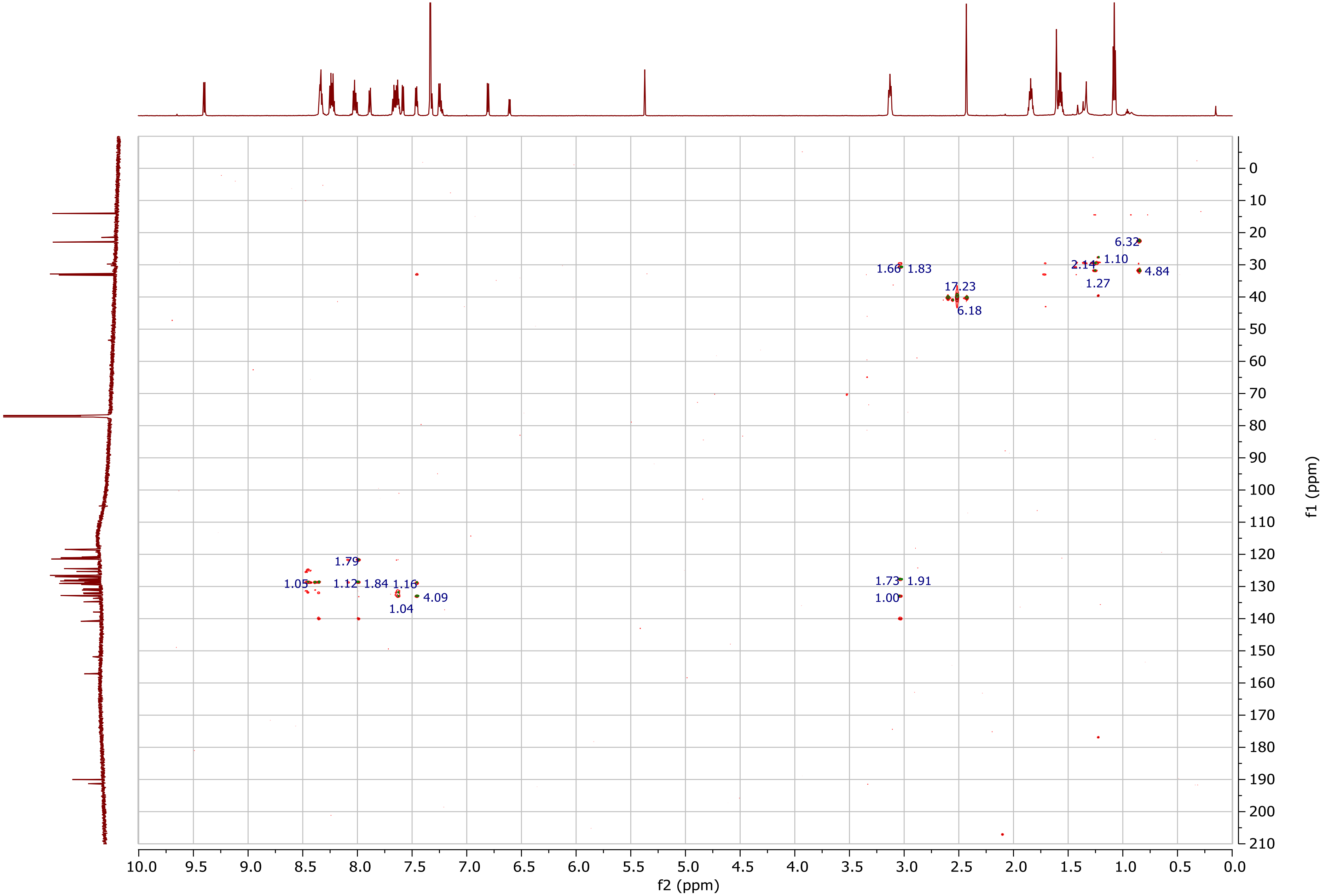

Supplement: Supplementary file 1 [file ijms-24-16483-s001.zip › ijms-2623956-supplementary.pdf]
